# Two Virus-Induced MicroRNAs Known Only from Teleost Fishes Are Orthologues of MicroRNAs Involved in Cell Cycle Control in Humans

**DOI:** 10.1371/journal.pone.0132434

**Published:** 2015-07-24

**Authors:** Brian Dall Schyth, Dennis Berbulla Bela-ong, Seyed Amir Hossein Jalali, Lasse Bøgelund Juel Kristensen, Katja Einer-Jensen, Finn Skou Pedersen, Niels Lorenzen

**Affiliations:** 1 National Veterinary Institute, Technical University of Denmark, Frederiksberg C, Denmark; 2 Fish Health Section, Department of Animal Science, Aarhus University, Aarhus N, Denmark; 3 Institute of Biotechnology and Bioengineering, Isfahan University of Technology, Isfahan, Iran; 4 Department of Molecular Biology and Genetics, University of Aarhus, Aarhus C, Denmark; 5 QIAGEN-AAR, Aarhus C, Denmark; Kunming University of Science and Technology, CHINA

## Abstract

MicroRNAs (miRNAs) are ~22 base pair-long non-coding RNAs which regulate gene expression in the cytoplasm of eukaryotic cells by binding to specific target regions in mRNAs to mediate transcriptional blocking or mRNA cleavage. Through their fundamental roles in cellular pathways, gene regulation mediated by miRNAs has been shown to be involved in almost all biological phenomena, including development, metabolism, cell cycle, tumor formation, and host-pathogen interactions. To address the latter in a primitive vertebrate host, we here used an array platform to analyze the miRNA response in rainbow trout (*Oncorhynchus mykiss*) following inoculation with the virulent fish rhabdovirus *Viral hemorrhagic septicaemia virus*. Two clustered miRNAs, miR-462 and miR-731 (herein referred to as miR-462 cluster), described only in teleost fishes, were found to be strongly upregulated, indicating their involvement in fish-virus interactions. We searched for homologues of the two teleost miRNAs in other vertebrate species and investigated whether findings related to ours have been reported for these homologues. Gene synteny analysis along with gene sequence conservation suggested that the teleost fish miR-462 and miR-731 had evolved from the ancestral miR-191 and miR-425 (herein called miR-191 cluster), respectively. Whereas the miR-462 cluster locus is found between two protein-coding genes (intergenic) in teleost fish genomes, the miR-191 cluster locus is found within an intron of a protein-coding gene (intragenic) in the human genome. Interferon (IFN)-inducible and immune-related promoter elements found upstream of the teleost miR-462 cluster locus suggested roles in immune responses to viral pathogens in fish, while in humans, the miR-191 cluster functionally associated with cell cycle regulation. Stimulation of fish cell cultures with the IFN inducer poly I:C accordingly upregulated the expression of miR-462 and miR-731, while no stimulatory effect on miR-191 and miR-425 expression was observed in human cell lines. Despite high sequence conservation, evolution has thus resulted in different regulation and presumably also different functional roles of these orthologous miRNA clusters in different vertebrate lineages.

## Introduction

MicroRNAs (miRNAs) are small (22–25 nucleotides long) non-coding RNA molecules that modulate gene expression in cells by pairing with complementary mRNAs to inhibit translation into proteins in a process called RNA interference (RNAi) [1]. Genes encoding cellular miRNAs are found in intergenic or intragenic regions in the genome and are transcribed by RNA polymerase II into 1–3 kb primary transcripts called primary miRNAs (pri-miRNAs) [2]. Pri-miRNAs, 5’-capped and poly-A tailed, fold into stem-loop-like structures, which are processed into precursor miRNA (pre-miRNA) by the type III ribonuclease Drosha and its co-factor Pasha (DiGeorge Syndrome Critical Region 8 Protein, DGCR8). Following export to the cytoplasm, the pre-miRNA is further processed into ~22 bp double stranded miRNA by another type III ribonuclease called Dicer together with TRBP (Tar RNA binding protein). Subsequently, the silencing strand of the miRNA is incorporated into and guides the multi-protein RNA-induced silencing complex (RISC) to seek out and bind target mRNAs. Target recognition is based on complementary base pairing between the 7-8-nucleotide-long seed region of the miRNA and the 3′ UTR region of the target mRNA. Binding of miRNA either inhibits translation or induce degradation of mRNA [3]. Since their first description in the nematode *Caenorhabditis elegans* [4], new miRNAs have been and are continuously being discovered. They are identified by a continuous numbering system and deposited in miRBase, which currently contains >30 000 miRNAs that have been annotated in 206 species (Release 20; http://www.mirbase.org/) [5, 6]. In humans, miRNAs have been shown to regulate the expression of >60% of protein coding genes [7]. A miRNA often target several genes, which may be involved in the same pathway or the same overall phenotype, thus augmenting the power and complexity of gene regulation by miRNAs [8]. Initially identified as a key regulator of development in *C*. *elegans* [3], miRNAs are now implicated in virtually all biological activities [9–14], including metabolism, cell cycle, immune responses and host-pathogen interactions [15–23].

Cellular miRNAs have been found to play roles in various viral infections in mammals [15, 18, 20–21]. A number of mammalian miRNAs have been revealed to participate in antiviral defense; either by directly targeting virus sequences to suppress viruses [24–29] or by regulating the expression of host genes that directly or indirectly negatively impact virus infection [30–33]. Some of these miRNAs have been shown to be induced by and to contribute to the antiviral effects of interferon (IFN) [24–25, 28, 32], the pivotal element in innate defense against viral pathogens in vertebrates [34]. Conversely, a non-canonical interaction between a host miRNA and the *Hepatitis C virus* genome enhances virus replication [35]. The ability of cellular miRNAs to modulate the expression of viral and immune-relevant host genes presents opportunities for understanding host-virus interactions and the design of novel antiviral therapies.

Although the roles of cellular miRNAs in host-virus interactions in mammals have been demonstrated, knowledge on the involvement of miRNAs in similar contexts in other vertebrates as yet remains scant. Additionally, whereas many miRNAs are conserved across vertebrate species, data on which genes they target and how they are regulated have appeared to be less consistent and their functional roles depend on the physiological specialization of individual vertebrate lineages [36–37]. We aimed to address these aspects in a teleost fish, a primitive vertebrate belonging to the most species-rich and phenotypically diverse group, which includes almost half of the extant vertebrate lineage [38]. We here analyzed the miRNA response using microarray in an economically important salmonid rainbow trout (*Oncorhynchus mykiss*), to the highly virulent fish rhabdovirus *Viral hemorrhagic septicaemia virus* (VHSV). VHSV belongs to the *Rhabdoviridae* family and the genus *Novirhabdovirus* [39]. It is the aetiological agent of viral hemorrhagic septicemia (VHS) that affects several fish species [40–46]. VHS is a highly significant disease in European aquaculture, severely impacting commercial production of rainbow trout because of massive economic losses that result from very high mortality rates during outbreaks [47]. Previous studies have shown that IFN and IFN-regulated genes are among the major elements in the early host response and contribute to protective immunity against VHSV [48].

We observed a very strong induction of two clustered miRNAs, miR-462 and miR-731 (herein referred to as the miR-462 cluster), in the liver of infected fish, as well as in fish cells stimulated by the TLR3 ligand and IFN inducer poly I:C. Analysis of regulatory sequences surrounding the miR-462 cluster locus revealed IFN-inducible and other immune-related promoter elements, suggesting involvement of these miRNAs in antiviral immune responses. Several miRNAs are highly conserved within vertebrates but miR-462 and miR-731 have only been described in teleost fishes according to miRBase. We therefore searched for their homologues in available vertebrate genomes. Gene synteny and sequence homology analyses showed that the teleost miR-462 and miR-731 are orthologues of miR-191 and miR-425, respectively, found in mammals as well as in the more primitive cartilaginous fish, elephant shark. In humans, the miR-191 cluster has been previously shown to be involved in cell cycle control and carcinogenesis, which appears unrelated to the involvement of their rainbow trout homologues in the response to VHSV infection and IFN stimulation. Here we describe for the first time how the two teleost fish virus-induced miRNAs differ from their human orthologues in terms of genome position and regulation and discuss how these differences may reflect functional specialization.

## Materials and Methods

### Viral challenge experiment

Two groups of 20 disease-free rainbow trout with an average weight of 5 g were kept in aquaria at 12°C (experimental temperature). Each aquarium contained 8 L of water, which was continuously renewed by a water flow-through system. The water flow-through was stopped and VHSV isolate DK-3592B [49] was added to one of the experimental aquaria (the other served as negative control) to give a final virus concentration of approximately 10^5^ TCID_50_/ml. After 1 h of viral challenge water flow-through was restarted. Fish were monitored daily for disease development for up to 14 days post-challenge (dpc). Diseased/moribund fish showing external signs of disease such as darkened skin, swollen belly (ascitis), swollen eyeballs (exophthalmia), and bleeding in eyes and skin, were terminated by lethal anaesthesia in benzocaine (ethyl p-aminobenzoate; Sigma cat.no. E-1501; diluted to 0.01%), counted, and recorded together with dead fish (also examined for external disease manifestations). Liver samples for RNA purification were taken from euthanized fish with external signs of disease 7 dpc (5 fish) and 8 (4 fish) dpc. At both times, the same number of fish was sampled from the negative control aquaria after being euthanized in benzocaine. Fish that survived the virus challenge, including unchallenged fish in negative control aquaria, were similarly terminated at the end of the experiment. All animal experiments were performed according to European and Danish rules for the use of experimental animals and approved by the Danish Committee for Animal Experiments (license no. 2007/561-1312). In approving the study protocol, the Danish Committee for Animal Experiments has considered that natural animal death without euthanasia may occur between monitoring points in virus challenge experiments aimed at mimicking a natural disease outbreak in fish and that some fish unintentionally die before proper diagnosis can be made due to the fast progression of disease. Verification of VHSV infection in diseased fish was carried out using a previously published ELISA on tissue samples [49].

### Total RNA purification from liver of diseased rainbow trout

Total RNA from the liver was isolated and purified using the miRNeasy Mini kit (Qiagen cat.no. 217004). RNA quantification and quality testing were conducted using the nanodrop system (Thermo Scientific, USA), followed by stabilization in RNA stable (Biomatrica, USA). All the steps in the procedure were as directed in the manufacturers’ protocol.

### Microarray analysis of miRNA regulation

Before microarray analysis, RNA samples were quality tested by verifying the presence of 28S rRNA, 18S rRNA and tRNA fractions after running for 1–2 hours (70 volts) in a 1% agarose gel containing 2% formaldehyde and stained using SyberGold (Invitrogen, USA). Furthermore, quantities and purity of samples were re-measured by nanodrop as above. The microarray for detecting miRNA levels was run by a microarray profiling service (Ocean Ridge Biosciences, USA). Microarray used the Ncode Version 2.0 probe set (Invitrogen) containing 1492 unique probes including 218 zebrafish probes (35-44-mer oligos spotted in triplicates) for detection of all miRNAs in the Sanger Institute database miRBase version 9.0 (Nov 2006). Two hundred ng RNA from each sample was labeled by ligation of DNA dendrimer containing 15-fluorophores to 3′-OH ends of miRNAs (Genisphere, USA). One-way ANOVA was used to assess the significance of the overall intensity difference between infected and non-infected fish. Raw data were log_2_ transformed and normalized to the mean signal of all detected probes (120 probes which were detectable both among the infected and control samples). Triplicate spots were averaged, throwing out flagged spots. Data from infected versus control samples for the 120 probe spots were compared using the NIA Array Analysis Software [50]. Fold regulation of individual miRNAs was calculated by dividing the mean intensity of signals from diseased fish with the mean intensity of signals from control fish. Raw microarray data are contained in S1 File.

### qPCR validation of miR-462 and miR-731 expression in the liver of VHSV-infected fish

1 μg of total RNA was used for cDNA synthesis by the QuantiMir Synthesis Kit (System Biosciences, USA, cat. no. RA420A-1) following manufacturer’s instructions. Briefly, RNAs were poly-A tailed using poly-A polymerase. The poly-A tail allows the binding of an oligo-dT adaptor containing a universal reverse primer binding site (anchor tail). Following reverse transcription from this adaptor, a cDNA pool consisting of anchor-tailed strands complementary to various RNAs in the original samples was generated.

qPCR for miRNAs was performed using the sequences of the mature dre-miR-462-5p and dre-miR-731-5p as forward primers together with the universal primer from the QuantiMir Synthesis Kit as reverse primer. Mature miRNA sequences can be found in miRBase (http://www.mirbase.org/) [5,6]. In this study, all mature sequences of the miRNAs of interest refer to the mature sequences that arise from the 5’ arm of the miRNA hairpin (i.e. miR-462-5p, miR-731-5p, etc). Furthermore, miR-462 and miR-731 refer to the miRNA sequences identified in rainbow trout, unless indicated otherwise.

Real-Time PCR was run on Mx3000P (Agilent Technologies, CA, USA) in reaction volumes of 25 μl containing a master mix solution (Brilliant SYBR Green QPCR Master Mix cat. no. 600548, Agilent Technologies, CA, USA), specific primers, ROX, water, and 5 μl of cDNA template diluted 100-fold. The program was set to 10 min at 95°C, 40 cycles of 20 sec at 94°C and 1 min at 60°C with collection of fluorescent data. Melting curves were determined by denaturing PCR products for 1 min at 95°C, followed by a ramp down to 55°C for 30 sec, and a gradual 0.2°C/sec climb to 95°C, continuously recording fluorescence. Transcript levels were measured as Ct values and normalized to omy-snoRNA-U23. PCR primer sequences are listed in S1 Table. Fold regulation was calculated from the mean values of duplicate measurements using the ΔΔC_*t*_ method, where fold regulation = 2^−ΔΔC^_*t*_ and ΔΔ*C*_*t*_ = (*C*_t miRNA infected_ − *C*_t reference gene_) − (C_t miRNA non-infected_ − *C*_t reference gene_). miRNA expression was analyzed in 6 VHSV-infected fish relative to 6 non-infected fish. The sizes of all PCR products were verified by inspection of the dissociation curve and by gel electrophoresis.

### Genomic localization of the miR-462 cluster and upstream region in available fish genome sequences

The locus containing miR-462 and miR-731 (miR-462 cluster) was localized on chromosome 8 of the zebrafish genome in Ensembl release 48 [51]. Synteny analysis was used to find homologous miRNA genes in all teleost fish genome assemblies present in Ensembl as well as in genomes of selected higher vertebrates, including human. This was done by searching *IMPDH2* and *DALRD3* genes flanking the locus that contains the miR-462 cluster in zebrafish. The miRNA loci were localized using mature miRNA sequences in NCBI blast2seq sequence alignment. Immunologically relevant promoter motifs upstream of the miRNA loci were found using the on-line database search tools Transcription Element Search System (TESS; http://www.cbil.upenn.edu/tess) and BIOBASE (www.biobase.de), as well as (blast2seq) (http://www.ncbi.nlm.nih.gov/) alignments for previously published motifs from fish. All searches were performed in the region 1000 nt upstream of the miR-462 cluster locus. Furthermore, mir-191 and mir-425 sequences from selected vertebrates (*Homo sapiens*, human; *Rattus norvegicus*, rat; *Bos taurus*, cow; *Mus musculus*, mouse; *Monodelphis domestica*, opossum; *Xenopus tropicalis*, Western clawed frog) were aligned with mir-462 and mir-731 sequences, respectively, from *Danio rerio* (zebrafish) in the CLC main Workbench (CLC, Denmark) using default settings. All miRNA sequences were retrieved from miRBase (http://www.mirbase.org/) [5, 6].

### Cloning and sequencing of the rainbow trout miR-462 cluster locus and its 5'- flanking region

Genomic DNA was extracted from the liver of disease-free rainbow trout using DNeasy Blood & Tissue Kit (Qiagen, Germany) following manufacturer’s instructions. DNA was eluted in TE Buffer (10 mM Tris·Cl, 0.5 mM EDTA, pH 9.0) and stored at -20°C. Three conserved sequences found by aligning fish genomic sequences were used to design primers that generate two amplicons. Amplicon 1 was generated using forward primer 5'-GTAACGGAACCCATAATGCAGCT-′3 and reverse primer 5'- CTTGGCTGACACGAAITTCCCGGT-'3. Amplicon 2 was generated with forward primer 5'- AGAAGTGAAAGTGAAA-3' and reverse primer 5'- CAGCTGCATTATGGGTTCCGTTAC- 3'. The two PCR products were cloned into TOPO TA Cloning Kit vector (Invitrogen). Plasmids were transformed in DH5α cells and cultured in LB medium with ampicillin (100 μg/ml) and screened for inserts by T7-M13 primers as specified by Invitrogen. Plasmids of positive clones were purified using the Plasmid DNA Purification kit (Qiagen). Inserts were verified by gel electrophoresis of 5' *EcoRI* and 3' *XbaI* double digests. Confirmed clones were sequenced by a commercial service provider (DNA Technology, Denmark) using the cloning primers. The sequence was deposited in the NCBI Genbank with accession number KP256534 (http://www.ncbi.nlm.nih.gov/nuccore/815871796).

### Construction of a reporter plasmid for monitoring promoter activity of the 5’-flanking region of the rainbow trout miR-462 cluster locus

The 5'-flanking region of the rainbow trout miR-462 cluster locus (containing the promoter area) was amplified by PCR. The forward primer (5'-GGAGATCTAGAAGTGAAAGTGAAATACA-3') corresponded to the sequence roughly 1000 nt upstream of the rainbow trout miR-462 cluster locus flanked by a 5′ terminal *Bgl*II restriction site (underlined). The reverse primer (5'-CGCTCTAGA TACCCGCTAACACCACTACTGAGT-3') corresponded to the opposite strand sequence immediately upstream the miR-462 cluster locus flanked by a *Xba*I restriction site in the 5' terminal (underlined). The following amplification conditions were used: 1 cycle of 94°C for 3 min; 14 cycles of 94°C for 60 sec, 54°C with a decrease of 0.5°C per cycle for 60 sec, 68°C for 2 min; 19 cycles of 94°C for 60 sec, 46°C for 60 sec, and 68°C for 1 min; and a terminal step of 5 min at 68°C. The PCR product (838 bp) was digested by *Bgl*II and *Xba*I after gel purification and cloned into pcDNA3.1/CT-GFP (Invitrogen) in which the CMV promoter has been excised by *Bgl*II and *Xba*I digestion followed by gel purification. The presence of the insert in the pcDNA3.1/CTprom-GFP plasmid was confirmed by restriction enzymes and by bidirectional sequencing (DNA Technology, Denmark). Plasmid concentration was determined using a Nanodrop spectrophotometer (Thermo Scientific, USA).

### Cell culture studies: transfection with reporter plasmid containing promoter of the 5’-flanking region of the mir-462 cluster locus, poly I:C stimulation, and virus challenge

For flow cytometry studies of promoter activation, 10^6^ RTS-11cells [52] were electroporated with 2μg pcDNA3.1/CT-ISREprom-GFP plasmid using the Microporator MP-100 system (Invitrogen, USA) for 24 well plates and 10 μl tips using two pulses of 1300 volts at a pulse width of 20. Immediately following electroporation, either 100 μg/ml poly I:C (Sigma-Aldrich GmbH, Steinheim, Germany; CAS # 42424-50-0) formulated 1:2 in N-[1-(2,3-Dioleoyloxy)propyl]-N,N,N-trimethylammonium methyl-sulfate (DOTAP, Roche) as previously described [49] or 20 μg/ml phytohaemagglutinin (PHA) was added directly to the medium. As controls, mock cells or cells transfected with plasmids without the presumed ISRE promoter or plasmid without EGFP were used. Following 48 hrs of stimulation at 15°C, cells were stained with CELL LAB ApoScreen Propidium Iodide (PI) (Beckman Coulter, USA) and run in a Beckman Coulter FC-500 flow cytometer. Graphs were prepared in the Kaluza software (Beckman Coulter, USA) gating out PI-stained dead cells

### Expression analysis of miRNAs and *ISG-12* gene in cell cultures

Cultured rainbow trout liver cells (RTL-W1) [53] were inoculated with VHSV with an MOI of approx. 10. Cells were harvested 24, 48, and 72 hrs post-infection. Human HeLa [54] and HEK293T [55] cells were stimulated with poly I:C formulated in DOTAP in final concentrations of either 5 μg/mL or 10 μg/mL. RTL-W1 cells were also stimulated with 10 μg/mL poly I:C (unformulated in DOTAP). RNA isolation, cDNA synthesis, and qPCR for miRNAs, were as described above. To detect human *ISG12* mRNA transcripts in HeLa and HEK293T cells, the human *ISG12* sequence was used as forward primer together with the universal primer from the kit, as above. Transcript levels were measured as Ct values and normalized to omy-snoRNA-U23 for omy-miR-462-5p and omy-miR-731-5p; either hsa-miR-16, hsa-let-7a, or hsa-snRNA U6 for hsa-miR-191-5p and hsa-miR-425-5p; and to hsa-18S rRNA for human *ISG-12* mRNA. We used the above mentioned reference genes for normalizing the levels of target transcripts because the expression of these normalizing genes has been determined to be stable in a separate experiment using the same cDNA samples. PCR primer sequences are listed in S1 Table. Fold regulation was calculated from the means of normalized Ct values of duplicate measurements from 3 cell culture wells using the ΔΔC_*t*_ method as described above.

### Bioinformatics analysis

Precursor and mature sequences of miR-191 and miR-462 cluster miRNAs were retrieved from miRBase Release 20 (http://www.mirbase.org/) [5, 6]. Teleost fish sequences were from *Oryzias latipes* (medaka), *Danio rerio* (zebrafish), and *Ictalurus punctatus* (Channel catfish) (except for *I*. *punctatus*, from which miRBase Release 20 does not have a record for mir/miR-731). Higher vertebrate sequences were from *Xenopus tropicalis* (Western clawed frog), *Anolis carolinensis* (Carolina anole, lizard), *Homo sapiens* (human), *Macaca mulatta* (macaque), *Pan troglodytes* (chimpanzee), *Pongo pygmaeus* (orangutan), *Gorilla gorilla*, *Cricetulus griseus* (Chinese hamster), *Bos taurus* (cow), *Equus caballus* (horse), *Ovis aries* (sheep), *Canis familiaris* (dog), *Ornithorhynchus anatinus* (platypus), *Monodelphis domestica* (opossum), *Sus scrofa* (pig), *Rattus norvegicus* (rat), *Mus musculus* (mouse), and *Taeniopygia guttata* (zebra finch) (exceptions: miRBase Release 20 does not have a record of mir/miR-191 from *T*. *guttata* and of mir/miR-425 from *G*. *gorilla*, *E*. *caballus*, and *O*. *aries*). Elephant shark mir/miR-191 and mir/miR-425 sequences were accessed from NCBI Nucleotide database (http://www.ncbi.nlm.nih.gov/nuccore/). Multiple sequence alignment was carried out on either stem loop or mature miRNA sequences using Clustal Omega through the European Molecular Biology Laboratory-European Bioinformatics Institute (EMBL-EBI) server (http://www.ebi.ac.uk/Tools/msa/clustalo/) [56]. The aligned miRNA sequences were then analyzed by Neighbour-Joining method using Jukes-Cantor nucleotide distance measure and 1000 replicates using CLC Genomic Workbench 7.0.3. The trees were subsequently visualized as unrooted neighbor joining (NJ) trees. Putative targets of hsa-miR-191 and hsa-miR-425 were predicted with the TargetScanHuman Release 6.2 algorithm, which predicts target mRNAs in vertebrate genomes (http://www.targetscan.org/vert_61/) [7]. Determination of the potential targets of miR-462 and miR-731 was performed using the TargetScanFish Release 6.2 algorithm, which predicts target mRNAs in the zebrafish genome (www.targetscan.org/fish_62/) [57].

## Results

### Identification of regulated miRNAs in the liver of VHSV-infected fish

**Fig 1:**
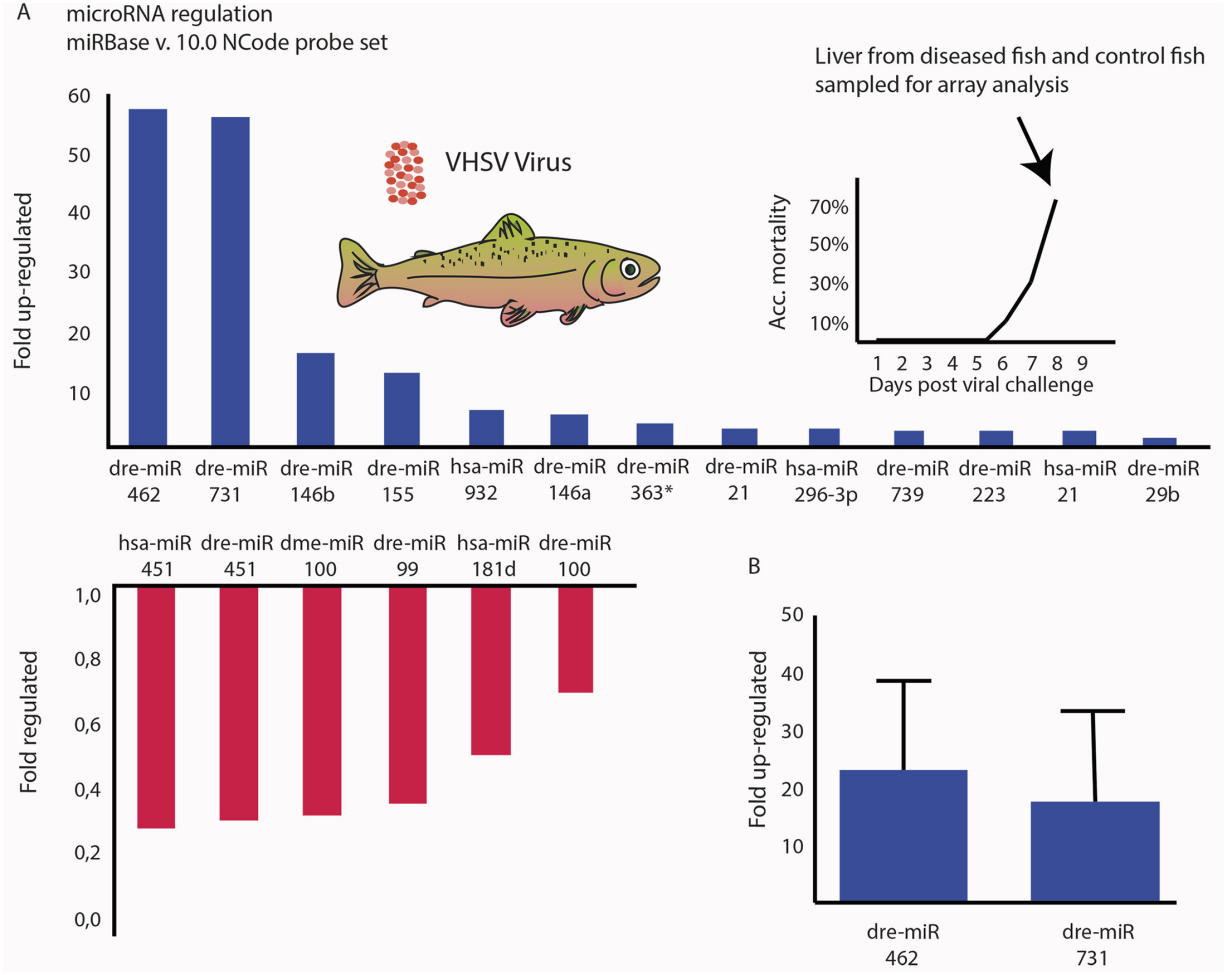
miR-462 and miR-731 are highly upregulated in the liver of VHSV-infected rainbow trout. (A) Microarray analysis of miRNA regulation in liver samples from diseased rainbow trout infected with *Viral hemorrhagic septicaemia virus* (VHSV). The Ncode V2 probe set covering miRBase v.9 was used to detect up-(blue) and down-regulated (red) miRNAs. The miRNA probes are denoted according to the miRBase nomenclature. Organisms: dre = *Danio rerio*, hsa = *Homo sapiens* and dme = *Drosophila melanogaster*. (B) qPCR validation of miR-462 and miR-731 expression in the liver of VHSV-infected rainbow trout. Fold regulation was calculated from the mean values of duplicate measurements using the ΔΔC_*t*_ method, using the omy-snoRNA U23 for normalization. miRNA expression was analyzed in 6 VHSV-infected fish relative to 6 uninfected fish.

Microarray was carried out in order to analyze which miRNAs are regulated in the liver of rainbow trout in response to infection with a fish rhabdovirus. The liver was chosen as a target because it is a central organ in the systemic innate response to infections. Microarray analysis (using 1492 unique miRNA specific probes) of the liver of VHSV-infected fish versus non-infected controls detected 120 miRNAs expressed significantly above the background level determined as mean of all probe signals. Of these, 13 showed more than two-fold up regulation in infected fish compared to controls (Fig 1A). Among these, miR-462 and miR-731 were particularly strongly upregulated (>50-fold). The strong expression levels of the two miRNAs were confirmed by qPCR, showing that miR-462 was upregulated ~20-fold and miR-731 ~15-fold (Fig 1B).

### Identification and characterization of an immune-induced polymerase type II promoter upstream of mir-462 cluster locus in fish

We found miR-462 and miR-731 particularly interesting as these have so far been described only in teleost fish. The mirBase currently lists miR-462 and miR-731 as only identified in zebrafish (*Danio rerio*) [58–59], medaka (*Oryzias latipes*) [60], Atlantic salmon (*Salmo salar*) [61] and channel catfish (*Ictalurus punctatus*) (where only miR-462 has been identified so far; [62]. The observation that these two miRNAs are most highly upregulated in VHSV-infected fish suggested their roles in host-virus interactions; yet studies on the involvement of miRNAs in the context of host-virus interactions in teleost fish is as yet limited. Therefore, we analyzed regulatory sequences in the vicinity of the miR-462 cluster locus in order to gain insight pertaining to the regulation of expression and to the potential role of miR-462 and miR-731 in teleost fish.

**Fig 2:**
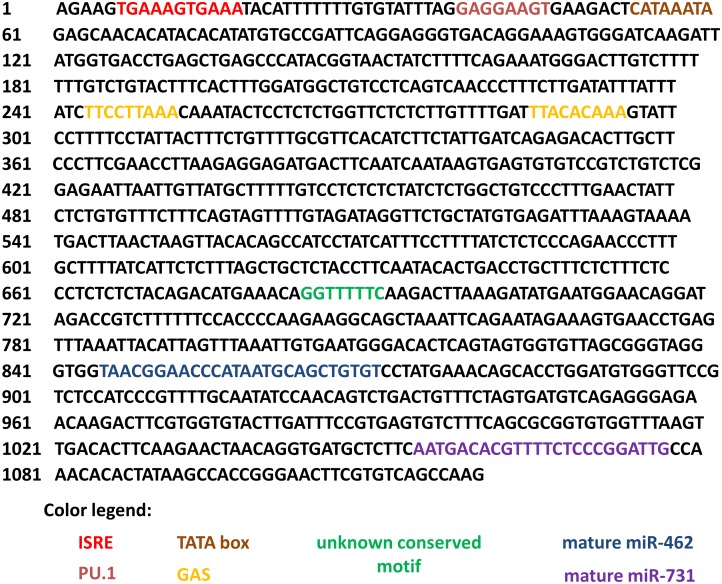
Analysis of presumed miR-462 cluster promoter region in the rainbow trout genome. The identified sequence is part of the intergenic area between *DALRD3* and *IMPDH2* in the trout genome and comprises 1119 bp. Several promoter motifs suggesting immunological regulation were identified including ISRE, PU.1, and GAS. Based on these motifs and the TATA box, the upstream sequence is presumed to be an IFN regulated class 2 polymerase promoter. The promoter motifs and the location between *DALRD3* and *IMPDH2* were found to be conserved among teleost fish.

A search in Ensembl database (http://www.ensembl.org/index.html) further showed that these miRNAs are found as an intergenic miRNA cluster between the housekeeping genes inosine monophosphate dehydrogenase 2 (*IMPDH2*) and DALR anticodon binding domain containing 3 (*DALRD3*) separated by 125 nucleotides and positioned on chromosome 8 in the zebrafish genome. We searched for *IMPDH2* and *DALRD3* in genomes of fish and selected higher vertebrates available in the Ensembl database and found evolutionary variants of the two miRNAs in all of them. In teleost fish genomes, the seed sequence and genome location for both mature miR-462 and miR-731 were found to be fully conserved. We aligned the sequences upstream of *IMPDH2* until *DALRD3* (which included the miRNA cluster locus) using the BLAST bl2seq and found some sequence motifs which are conserved in teleost fish. We used primers recognizing the conserved sequence motifs together with conserved sequences in the premature miRNAs to sequence a 1000-bp stretch of the rainbow trout genome flanking the 5′-side of the cluster upstream the mir-731 hairpin gene (Fig 2). We searched this sequence using the Transcription Element Search System (TESS; http://www.cbil.upenn.edu/tess) and BIOBASE immune specific binding sites prediction (http://www.biobase.de) and identified an IFN-stimulated response element (ISRE, G/A/T + GAAANNGAAA + G/C + A/T/C) [63–66] 828 bp upstream of the start of the mir-462 locus (Fig 2), indicating type I IFN inducibility. This motif is 100% conserved among teleost fishes (S1 Fig), indicating conserved regulation. Another motif, the purine box 1 (PU.1: GAGGAAGT) involved in the development of granulocytes, macrophages, and lymphocytes in jawed vertebrates [67–68], was similarly conserved among teleosts (S1 Fig). In the rainbow trout sequence, we further identified TATA element [69], indicative of RNA polymerase II-dependent expression; and a motif which was repeated twice and resembled the gamma interferon activated site (GAS) element (IFN-gamma activated sequence; TTN CNN NAA) [70–72]. In addition, the sequence of unknown promoter involvement, GGTTTTTTC, was also found to be conserved in other fish genomes (Fig 2).

### Identification of miR-462 and miR-731 orthologues in mammalian genomes

**Fig 3:**
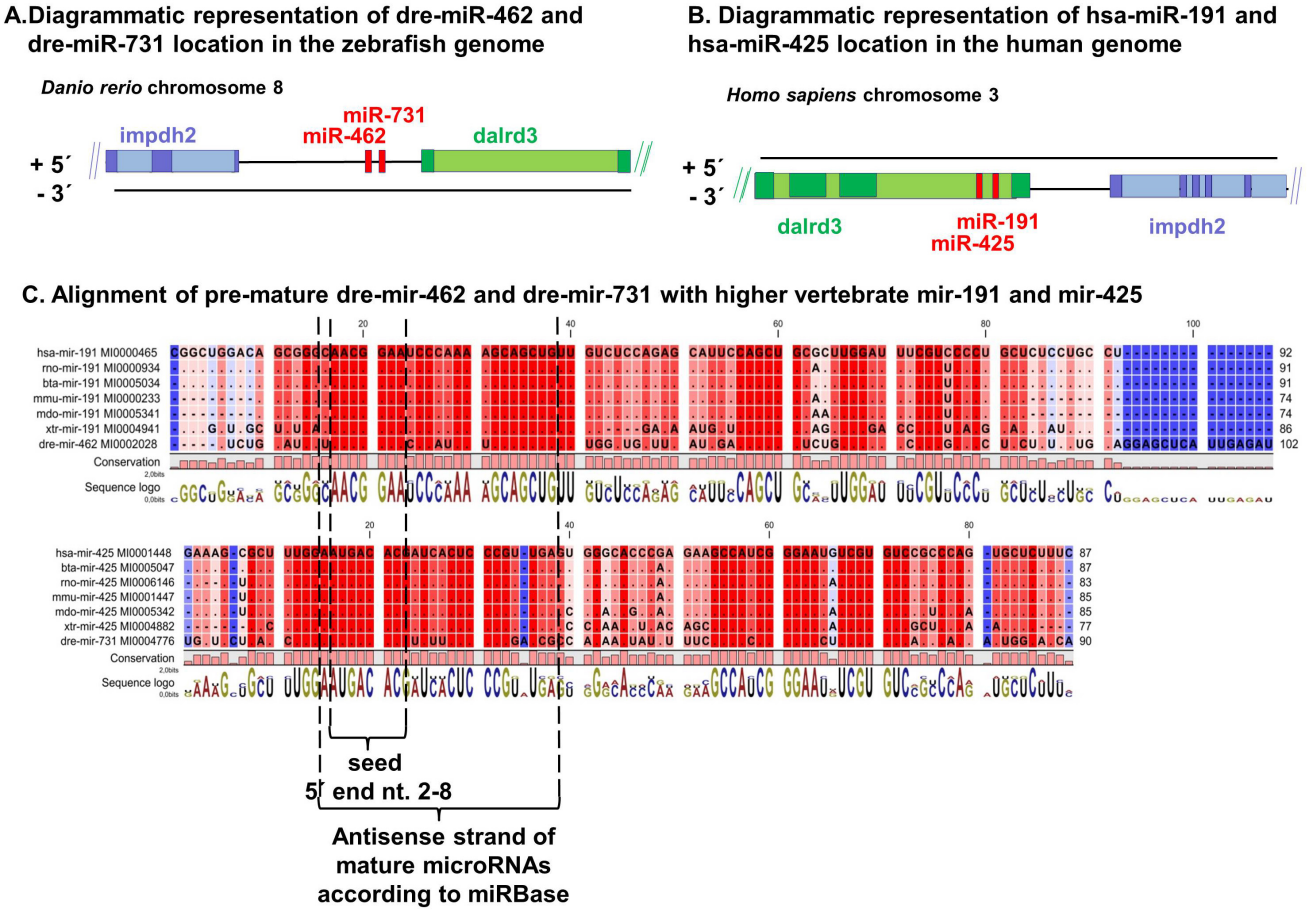
The miR-462 and miR-731 in teleost fish are orthologues of miR-191 and miR-425, respectively, in higher vertebrates. Diagram of the genomic location of the (A) miR-462 cluster in zebrafish and (B) the miR-191 cluster in humans. Although the diagram represents the zebrafish and human genome regions, the location of the miRNA clusters is similar to that in other teleost fish genomes and some higher vertebrate genomes, as discussed in the text. (C) Alignment of pre-miRNA sequences from various vertebrate species. The location of the mature antisense miRNA including the conserved seed region is highlighted below the alignments. hsa = *Homo sapiens* (human), bta = *Bos taurus* (cow), rno = *Rattus norvegius* (rat), mmu = *Mus musculus* (mouse), mdo = *Monodelphis domestica* (opossum), xtr = *Xenopus tropicalis* (frog), and dre = *Danio rerio* (Zebrafish). See S2 Fig for an alignment including more species.

Because several miRNAs are highly conserved among the vertebrates, we were interested to find potential homologues of the two teleost fish miRNAs in available vertebrate, particularly mammalian, genomes. Synteny analysis between teleost fish and human genomes showed two intragenic/intronic miRNAs, miR-191 and miR-425, within the region flanked by *DALRD3* and *IMPDH2* instead of the intergenic miR-462 and miR-731 present in teleost fish genomes analyzed (http://www.ensembl.org/index.html) (Fig 3A and 3B). Alignment of miR-191 and miR-425 with miR-462 and miR-731, respectively, showed high homology especially in the antisense strand and a full conservation in the seed area (Fig 3C). Furthermore, by aligning the fish and the human *DALRD3* sequences, we found that the miR-191 cluster sequences were absent in the fish *DALRD3* locus (data not shown). Together, these findings suggest that the intergenic teleost fish miR-462 cluster is phylogenetically related to the intragenic miR-191cluster. Interestingly, while comparing several mammalian genomes from the Ensembl database, we noted that whereas the human, macaque, and marmoset miR-191 cluster is present intragenically in an intron of *DALRD3* (Fig 3A and 3B), this is not the case for all mammalian genomes.

### Stimulation of miRNA expression in cell culture

**Fig 4:**
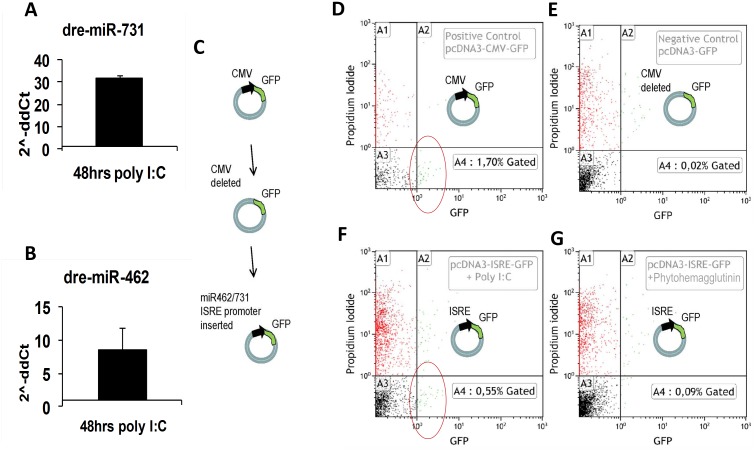
Expression of miR-462 and miR-731 can be activated by the TLR3 ligand poly I:C. (A-B) Rainbow trout liver cells RTL-W1 in culture up-regulated miR-731 and miR-462 following stimulation with poly I:C. (C-G) Transfection with a mir-462 cluster promoter- GFP-reporter plasmid construct cells followed by stimulation with poly I:C, induced GFP expression in rainbow trout RTS-11 cells (F). Red circles in flow cytometry diagrams indicate cells with green fluorescence higher than the gated negative cells. Cells transfected with a positive control GFP-reporter plasmid containing the CMV promoter (D) and a negative control plasmid GFP reporter without any promoter (E) were used to gate for GFP positive cells (quadrant A4) and GFP negative cells (quadrant A3) respectively. Red dots (quadrant A1) and green dots (quadrant A2) indicate dead cells according to their staining above background with propidium iodide. Such cells were not considered in the analysis. No up regulation was seen in cells following stimulation with the TLR-4 ligand phytohaemagglutinin (PHA) (G).

The presence of IFN-relevant promoter elements upstream of the mir-462 locus in fish genomes and the non-association of these with the miR-191 locus in the human genome indicate potential regulation of miR-462 and miR-731, but not of miR-191 and miR-425, by IFNs. We thus wanted to find out whether the expression of miR-462 and miR-731 (in fish cells) and miR-191 and miR-425 (in human cells) will be induced in response to stimulation with the TLR3 agonist and general IFN stimulator poly I:C. Furthermore, we were interested to know if the rainbow trout mir-462 promoter containing IFN-relevant motifs will be able to drive the expression of a reporter gene following poly I:C simulation of cells. Stimulation of rainbow trout liver cells (RTL-W1) with poly I:C upregulated the expression of miR-462 and miR-731 (Fig 4A and 4B). On the other hand, infection of RTL-W1 cells with VHSV did not stimulate an upregulation of the miRNAs but rather caused downregulation (S3 Fig). The presumed miRNA promoter sequence was checked for its ability to promote the expression of the green fluorescent protein (GFP) in rainbow trout macrophage cell line (RTS-11) transfected with a GFP reporter plasmid construct carrying the suspected miRNA promoter sequence upstream of the GFP gene. Flow cytometry analysis confirmed that these cells could be stimulated by poly I:C to express GFP, while the IFN-γ-stimulating ligand phytohemagglutinin (PHA) failed to induce GFP expression (Fig 4C–4G).

Stimulation of HeLa cells with poly I:C induced the expression of the IFN-induced gene *ISG12* but not miR-191 and miR-425 (S4 Fig). We used HEK293T cells as control as these are known not to be poly I:C inducible due to lack of TLR3 expression [73–74] and showed neither *ISG12* regulation nor significant regulation of the examined miRNAs.

### Phylogenetic analysis

Based on the high homology of the seed regions in the non-teleost miR-191 and teleost miR-462 clusters (Fig 3C), conserved clustering pattern, related genomic location as well as lack of genomic co-existence, we hypothesized these miRNA clusters to represent evolutionary orthologues. We therefore performed phylogenetic analysis based on the mature sequences of the miR-191 and miR-462 cluster miRNAs retrieved from miRBase to elucidate their evolutionary history in vertebrates.

**Fig 5:**
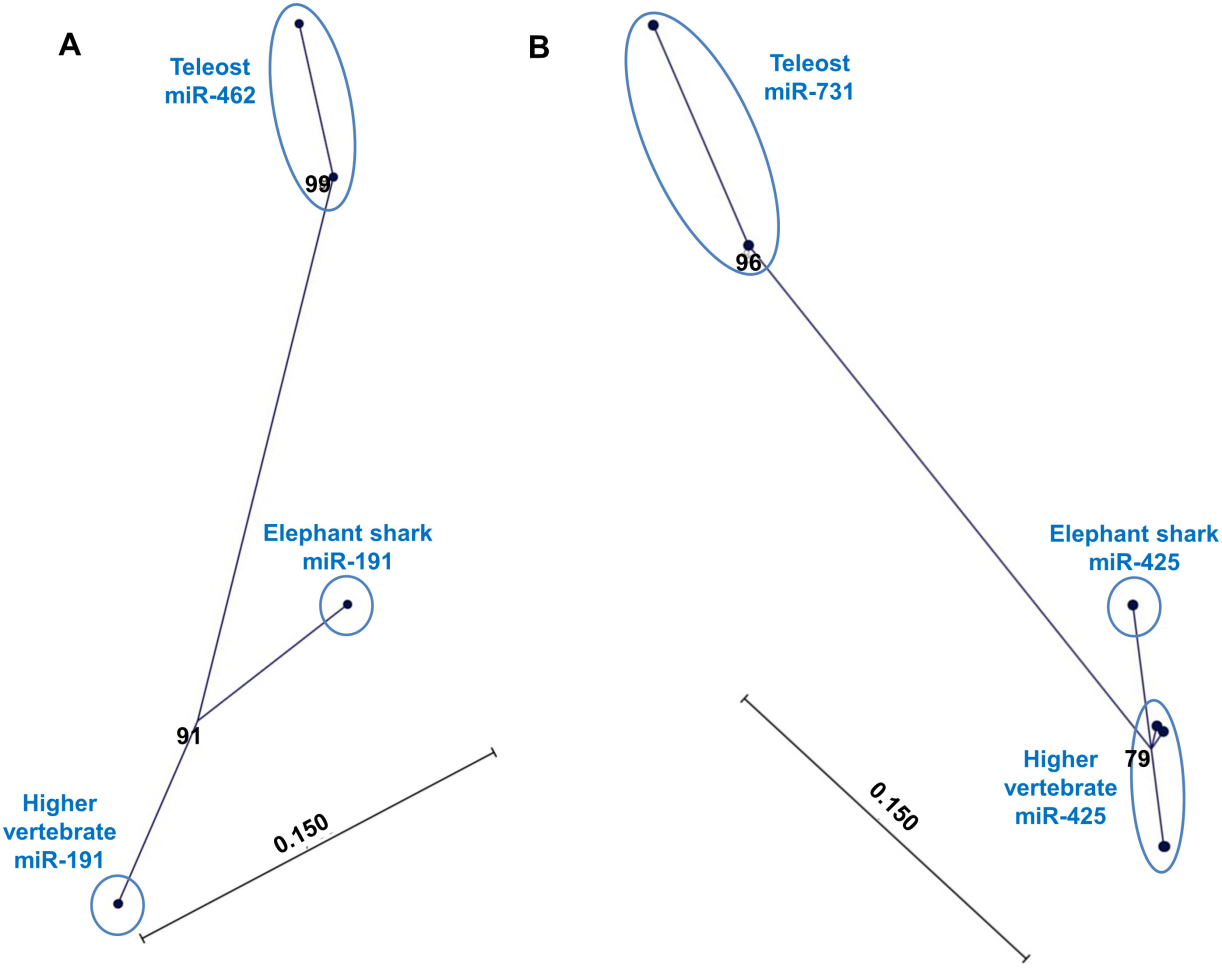
Teleost miR-462 and miR-731 have evolved from the ancestral miR-191 and miR-425, respectively. Phylogenetic analysis of (A) miR-191 and miR-462 and (B) miR-425 and miR-731. Unrooted neighbor-joining (NJ) trees with bootstrap values from 1000 replicate analysis indicated at the nodes as percentage values. In 5B, high similarity among the sequences originating from higher vertebrates makes the end nodes appear overlapping, thereby mimicking a single node. The scale bar represents genetic distance.

Unrooted neighbor-joining (NJ) bootstrapped distance trees (1000 replicates) confirmed that the teleost miR-462 and miR-731 clustered together with the non-teleost miR-191 and miR-425, respectively. However, the teleost subclades were distinct from those comprising the higher vertebrates, with the cartilaginous fish (elephant shark; *Callorhinchus milii*) clustered in between (Fig 5A and 5B). On the other hand, miR-191 and miR-425 of diverse higher vertebrates all clustered together and were thus almost identical. The genetic distance shown in the phylogenetic tree, in terms of the number of mutation/evolutionary events between species since their divergence (calculated using the Jukes-Cantor model), indicates the extent of divergence of the teleost miRNAs from those of higher vertebrates and that of the Elephant shark.

## Discussion

The data presented here are the first functional and evolutionary analyses of a cluster of two virus-induced miRNAs (miR-462 and miR-731) found only in teleost fishes. MicroRNAs are endogenous small RNA molecules which have appeared to play an important role in posttranscriptional regulation of protein expression in many living organisms [1,75]. Their high level of conservation across vertebrate species suggests involvement in generic biological processes but is not consistently supported by currently available data [36–37]. We report here that miR-462 and miR-731 in teleost fishes are orthologous, respectively, to miR-191 and miR-425 present in other vertebrates, but differ in the regulation of their expression and detailed genomic position. The results suggest that the miRNA clusters have evolved different functional roles in different vertebrate lineages.

Based on teleosts belonging to an evolutionary early vertebrate lineage, we initially thought that the miR-462 cluster represented an ancestral form of the mammalian miR-191 cluster. However, the recently discovered presence of the miR-191 cluster in the cartilaginous fish *C*. *milii* or elephant shark, which has been claimed to have the slowest evolving genome of all known vertebrates [76], suggested that the miR-191 cluster has existed in the common ancestor of cartilaginous fishes and bony vertebrates. The probability of convergent evolution of two miRNAs is considered to be small [77–78] and animal miRNAs generally exhibit a low rate of secondary loss [79]. Together with their consistent position relative to the *DALRD3* locus as well as the association of the two miRNAs comprising the given miRNA cluster in all genomes analyzed, this further supports the genetic relation of the miR-462 and miR-191 clusters.

Phylogenetic analysis accordingly demonstrated that the teleost fish miRNA cluster represented a distinct subclade within their non-teleost homologues. This basically suggests that the fish miR-462 and miR-731 should be considered as orthologues of the non-teleost miR-191 and miR-425, respectively. The larger genetic distance (Fig 5) that separates the teleost fish miR-462 cluster from the orthologous miR-191 cluster of other vertebrates indicates that teleost miRNA sequences in relative terms have undergone more accelerated changes. The most important evolutionary adaptation within the teleost fish appeared to be the local regulatory elements outside the miRNA cluster, as discussed below.

All available teleost fish genomes have the miR-462 and miR-731 loci positioned in an intergenic region between the *DALRD3* and *IMPDH2* loci whereas humans and some other mammals have the homologous miR-191 cluster within *DALRD3* [51]. The intergenic position of the miR-191 cluster in the genome of the primitive cartilaginous fish, *C*. *milii* [76], suggests that a translocation of the miR-191 locus into the *DALDR3* intron has occurred during vertebrate evolution in the primate lineage including human, macaque, and marmoset.

Regulatory elements found upstream of the teleost miR-462 cluster locus suggested the involvement of the miRNAs in innate immune functions. While the TATA-box indicated that the expression of miR-462 and miR-731 is steered by a polymerase type II promoter as reported for other miRNAs [2], the presence of ISRE, GAS and PU.1 elements appeared unique for teleosts. These elements themselves are rather conserved among vertebrates [66–68, 80–81], but are not associated with the miR-191 cluster in mammals. The ISRE element is a binding site for IFN-regulatory factors (IRFs) and IFN-stimulated gene factor 3 (ISGF3). Increased expression of the miR-462 and miR-731 in rainbow trout RTL-W1 liver cells stimulated with the TLR3 ligand poly I:C, known as a strong inducer of type 1 IFN, further supported a regulatory role of the latter (Fig 4A and 4B). The observed downregulation of miR-462 cluster miRNAs in VHSV-infected RTL-W1 cells (S3 Fig) was contradictory to what was observed in the fish, but correlated with a lack of IFN induction in the RTL-W1 cells following VHSV infection. This probably reflects that VHSV is able to suppress IFN induction in the individual infected cell [82], as well as in cell cultures given a high inoculum leading to simultaneous infection of all cells. The presence of the GAS element indicates IFN-γ-regulated transcription. However, attempts to induce miR-462 and miR-731 expression by stimulation of RTS-11 monocyte cells with the traditional IFN-γ inducers PHA and LPS did not give a detectable signal (Fig 4G), despite the RTS-11 cells having been shown to upregulate levels of IFN-γ-inducible protein 10 (IP-10), as well as the key IFN-γ intracellular signalling molecule STAT 1 in response to PHA stimulation [83]. Similarly, no signal related to the promoter activity of the PU.1 binding sequence (PU.1 box) was observed. In rainbow trout, the leukocyte differentiation related transcription factor PU.1 is known to be upregulated tissue specifically in the muscle, liver, intestine, brain, head kidney, and in macrophages in primary cell culture following LPS stimulation [80]. The lack of stimulation of reporter gene expression by PHA and LPS in our setup remains to be explained but it might be that the cloned promoter region is incomplete in terms of motifs required to respond to those agents. We did not perform further analysis of the promoter activity of the upstream region of the miR-191 cluster locus since this region in mammalian genomes does not contain IFN-related elements and has no similarity to that found upstream of the teleost miR-462 cluster locus; and poly I:C stimulation of HeLa cells accordingly did not result in the induction of miR-191 and miR-425 expression.

While IFN-induced expression of miRNAs as well as the involvement of miRNAs in antiviral immune mechanisms in mammals has been described earlier [24–33], this is to our knowledge the first report describing the involvement of the teleost miR-462 and miR-731 in IFN-mediated response to virus infection. Similar findings have not been reported for the human orthologues, although expression of miR-191 in colonic mucosal tissue of inflammatory bowel disease patients [84] and its higher levels in peripheral blood of Crohn’s disease patients relative to healthy individuals [85] suggests a potential immunological involvement. In addition, miR-191 and miR-425 expression was upregulated by IL-2 stimulation of human natural killer (NK) cells [86]. Although the functional impact of the miR-191 cluster miRNAs remains unclear in the above-mentioned studies, the results indicate this miRNA cluster’s potential role in inflammatory responses. A recent report further demonstrated involvement of miR-425 in inflammation-induced cancer, which together with miR-191 and other miRNAs, has been shown to be upregulated in IL-1β-treated human gastric adenocarcinoma cell line AGS [87]. Reduced cell proliferation and migration following inhibition of miR-191 and miR-425 in a gastric cancer cell line further supported their involvement in oncogenesis and cell cycle control [88]. As expected, we were not able to induce miR-191 nor miR-425 in the human HeLa cells with poly I:C despite the high up-regulation of the IFN-stimulated gene *ISG-12*. In addition, miR-191 and miR-425 were not among the many inducible miRNAs found in a virus replicon-bearing human hepatoma cell line, nor were they upregulated by the addition of ribavirin and/or IFN-β to these cells [89]. The regulation of the intragenic miR-191 cluster in humans thus appears to be different, possibly controlled by proinflammatory signaling molecules like IL-1β [87], estrogen response elements [90–91], other regulatory elements [92–93], and by epigenetic mechanisms [94]. Furthermore, the implication of the human miR-191 cluster in various physiological processes [95–99] and in a number of pathologies and human neoplasias [87–88, 90–94, 100] indicate a wide and diverse expression pattern, target repertoire, and regulatory roles in diverse processes in multiple organs and tissues. As discussed by Ason at al. [37], differences in miRNA expression profiles—and thereby differences in regulation—between vertebrate species increases with increased physiological differences. Accordingly, Xu et al. [36] found a very high evolvability of miRNA target sites between fish and humans and low evolvability between chimpanzee and human using cross-linking immunoprecipitation data.

In conclusion, our study showed that the IFN-regulated miR-462 and miR-731 in teleost fishes are orthologues, respectively, of the ancestral vertebrate miR-191 and miR-425 present in a wide range of non-teleost vertebrates from primitive cartilaginous fish to humans but that the teleost variant has evolved differently with respect to its regulation and presumably also functional roles. In contrast to the miR-462 cluster in teleost genomes, the human miR-191 cluster does not appear to be involved in IFN-mediated immunological functions but may play a role in non-IFN associated inflammatory responses and cell proliferation. Thus, the regulatory changes through evolution of the orthologous miRNA clusters likely reflect functional specialization/diversification in different vertebrate lineages. This was supported by our *in silico* target prediction analysis suggesting very different target profiles in fish and humans (S2–S5 Tables) but requires further functional analysis as well as target sequence validation. To date, validated targets for miR-462 and miR-731 are not known. Validated targets for miR-191 in humans include genes encoding transcription factors, chromatin remodelers, and regulators of the cell cycle [101] and for miR-425 genes encoding a tumor suppressor [87] and the atrial natriuretic peptide involved in salt intake response [96]. Our findings support earlier analyses showing that miRNA sequence conservation among distantly related species may not necessarily imply functional conservation, particularly between species with larger differences in physiology [37]. The evolution of IFN-related response elements associated with the miR-462 cluster in teleost fishes seems to reflect some unique features of the innate immune response in teleost fishes. Specialization in the function of these miRNAs across this highly diverse vertebrate group could have resulted from the need to diversify innate immune defence strategies as an adaptation to various aquatic habitats with varying temperatures and frequent exposure to water-borne viruses. In mammals, host miRNAs induced by virus infections have been found to target either host or viral genes—or both, and their effect can be advantageous for either the host or the virus [3, 15, 24–33]. Further functional studies are expected to reveal how the teleost miR-462 and miR-731 are involved in the response to virus infection in teleost fish. Accordingly, our most recent data thus suggest that these two miRNAs contribute to the IFN-related innate protection as induced by the TLR3 agonist poly I:C [102]. Very recently, the miRNA response to another fish rhabdovirus, *Spring viremia of carp virus* (SVCV), was studied in cell culture, and among many others, miR-462 and miR-731 were found only to be moderately upregulated [103]. However, *in vivo* experiments will be needed to determine how these findings relate to our observations.

## Supporting Information

S1 Fig Conservation of specific sequence motifs upstream miR-462 and miR-731. The area upstream of the two miRNAs (-1000nt) were retrieved from selected fish genomes (stickleback, *Gasterosteus aculeatus*; medaka, *Oryzias latipes*; green spotted puffer, *Tetraodon nigroviridis*; and zebrafish, *Danio rerio*) in the UCSC database. By alignment of these sequences conserved motifs were found which were later identified as ISRE and PU.1.
(TIF)

S2 Fig Alignment of dre-mir-462/731 with higher vertebrate mir-191/425 stem loops (referring to Fig 3). ola = *Oryzias latipes* (medaka); dre = *Danio rerio* (zebrafish); ipu = *Ictalurus punctatus* (Channel catfish); xtr = *Xenopus tropicalis* (Western clawed frog); aca = *Anolis carolinensis* (Carolina anole, lizard); hsa = *Homo sapiens* (human); mml = *Macaca mulatta* (macaque); ptr = *Pan troglodytes* (chimpanzee); ppy = *Pongo pygmaeus* (orangutan); ggo = *Gorilla gorilla*; cgr = *Cricetulus griseus* (Chinese hamster); bta = *Bos taurus* (cow); eca = *Equus caballus* (horse); oar = *Ovis aries* (sheep); cfa = *Canis familiaris* (dog); oan = *Ornithorhynchus anatinus* (platypus); mdo = *Monodelphis domestica* (opossum); ssc = *Sus scrofa* (pig); rno = *Rattus norvegicus* (rat); mmu = *Mus musculus* (mouse); tgu = *Taeniopygia guttata* (zebra finch). *Callorhinchus milii* (elephant shark) sequences are indicated by gi/511677982 and gi/511678023.
(TIF)

S3 Fig Rainbow trout liver cells RTL-W1 in culture did not up-regulate miR-731 and miR-462 24, 48, and 72 hrs following infection with VHSV. (TIF)

S4 Fig Human miR-191 cluster regulation is not steered by a poly I:C activated promoter. (A-C) Immune stimulation by poly I:C does not induce upregulation of miR-191 in HeLa cells. Normalization to hsa-miR-16 (A) showed a tendency towards regulation following poly I:C stimulation but was not significant. Normalization of the same expression data to hsa-let-7a (B) and hsa-snRNA U6 (C) did also not show any significant changes. Cells were either mock treated with DOTAP or treated with DOTAP-formulated poly I:C at 5 or 10 ug/ml concentrations. The negative control (NK) consisted of untreated cells. Standard deviations are shown. (D) Despite no significant upregulation of hsa-miR-191 following poly I:C stimulation, poly I:C treatment induced a strong concentration-dependent interferon response, as shown by the upregulation of ISG12 in Hela cells. Note that the values on the y-axis in (D) are much higher than in (A-C). The samples were also checked for the regulation of hsa-miR-425, which also showed no significant regulation (data not shown). Human embryo kidney cells (HEK293T) were used as negative control cells because an innate cellular response in these cells cannot be induced by poly I:C. Accordingly, these neither regulated ISG12 nor miR-191/miR-425 (data not shown).
(TIF)

S1 File Microarray data using the Ncode V2 probeset covering miRBase v.9. (XLS)

S1 Table Primers used in qPCR experiments. (DOCX)

S2 Table Selected putative targets of miR-191 in vertebrate genomes predicted using the TargetScan Release 6.2 algorithm and ranked by their probability of conserved targeting (P_CT_). (Only top-ranked targets are shown.)
(DOCX)

S3 Table Putative targets of miR-425 in vertebrate genomes predicted using the TargetScan Release 6.2 algorithm and ranked by their probability of conserved targeting (P_CT_). (DOCX)

S4 Table Putative targets of miR-462 in the zebrafish genome predicted using the TargetScanFish Release 6.2 algorithm and ranked by their probability of conserved targeting (P_CT_). (DOCX)

S5 Table Putative targets of miR-731 in the zebrafish genome predicted using the TargetScanFish Release 6.2 algorithm and ranked by their probability of conserved targeting (P_CT_). (DOCX)
