# Supplementary figures and images for: Two Virus-Induced MicroRNAs Known Only from Teleost Fishes Are Orthologues of MicroRNAs Involved in Cell Cycle Control in Humans
Source: PLoS One. 2015 Jul 24;10(7):e0132434. doi: 10.1371/journal.pone.0132434 (PMC4514678; doi:10.1371/journal.pone.0132434)

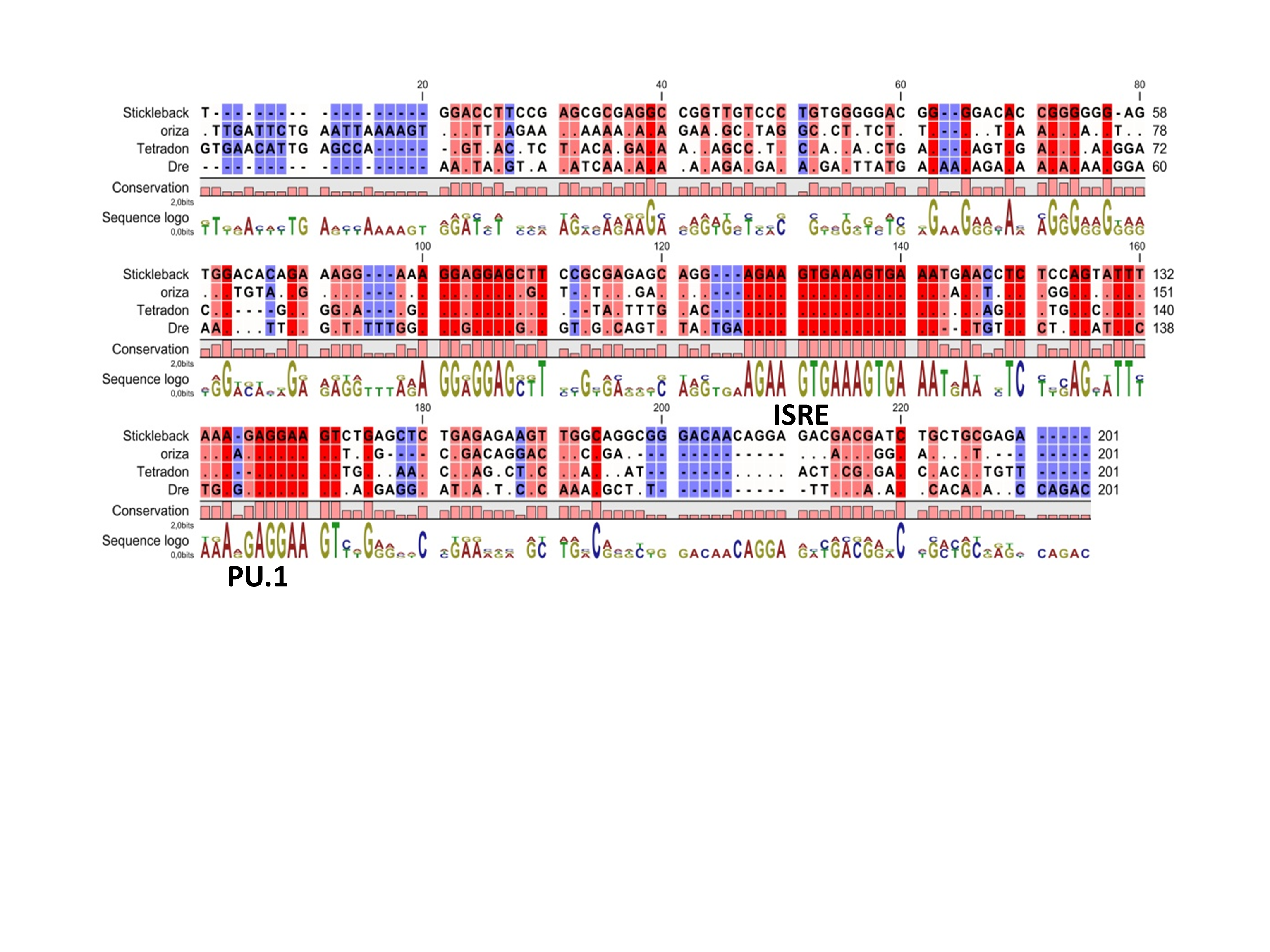

Supplement: S1 Fig — The area upstream of the two miRNAs (-1000nt) were retrieved from selected fish genomes (stickleback, Gasterosteus aculeatus; medaka, Oryzias latipes; green spotted puffer, Tetraodon nigroviridis; and zebrafish, Danio rerio) in the UCSC database. By alignment of these sequences conserved motifs were found which were later identified as ISRE and PU.1. (TIF) [file pone.0132434.s001.tif]

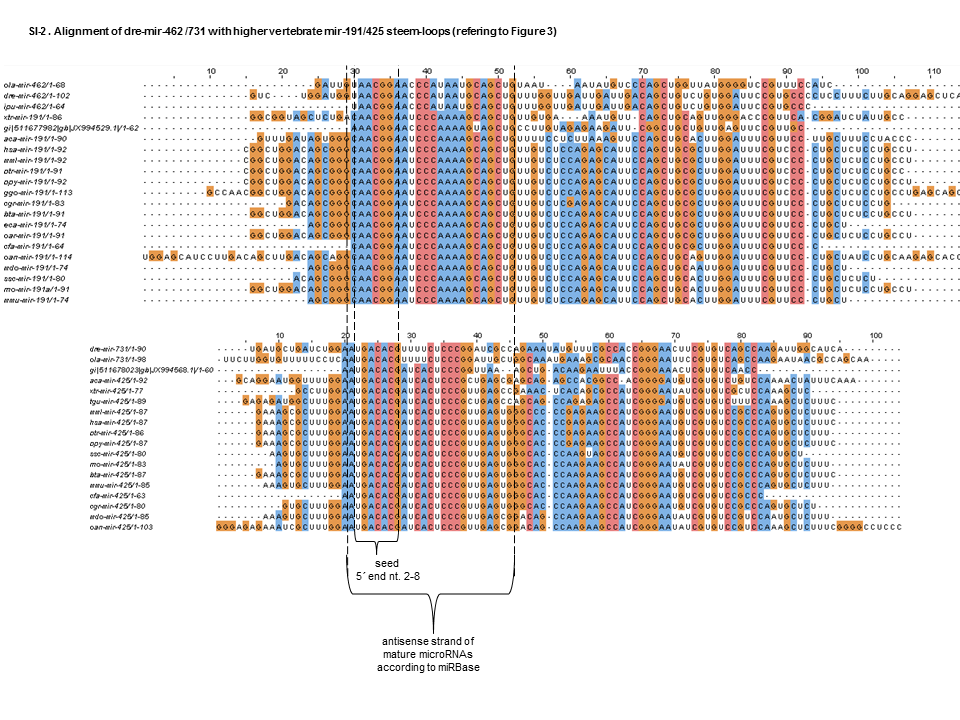

Supplement: S2 Fig — ola = Oryzias latipes (medaka); dre = Danio rerio (zebrafish); ipu = Ictalurus punctatus (Channel catfish); xtr = Xenopus tropicalis (Western clawed frog); aca = Anolis carolinensis (Carolina anole, lizard); hsa = Homo sapiens (human); mml = Macaca mulatta (macaque); ptr = Pan troglodytes (chimpanzee); ppy = Pongo pygmaeus (orangutan); ggo = Gorilla gorilla; cgr = Cricetulus griseus (Chinese hamster); bta = Bos taurus (cow); eca = Equus caballus (horse); oar = Ovis aries (sheep); cfa = Canis familiaris (dog); oan = Ornithorhynchus anatinus (platypus); mdo = Monodelphis domestica (opossum); ssc = Sus scrofa (pig); rno = Rattus norvegicus (rat); mmu = Mus musculus (mouse); tgu = Taeniopygia guttata (zebra finch). Callorhinchus milii (elephant shark) sequences are indicated by gi/511677982 and gi/511678023. (TIF) [file pone.0132434.s002.tif]

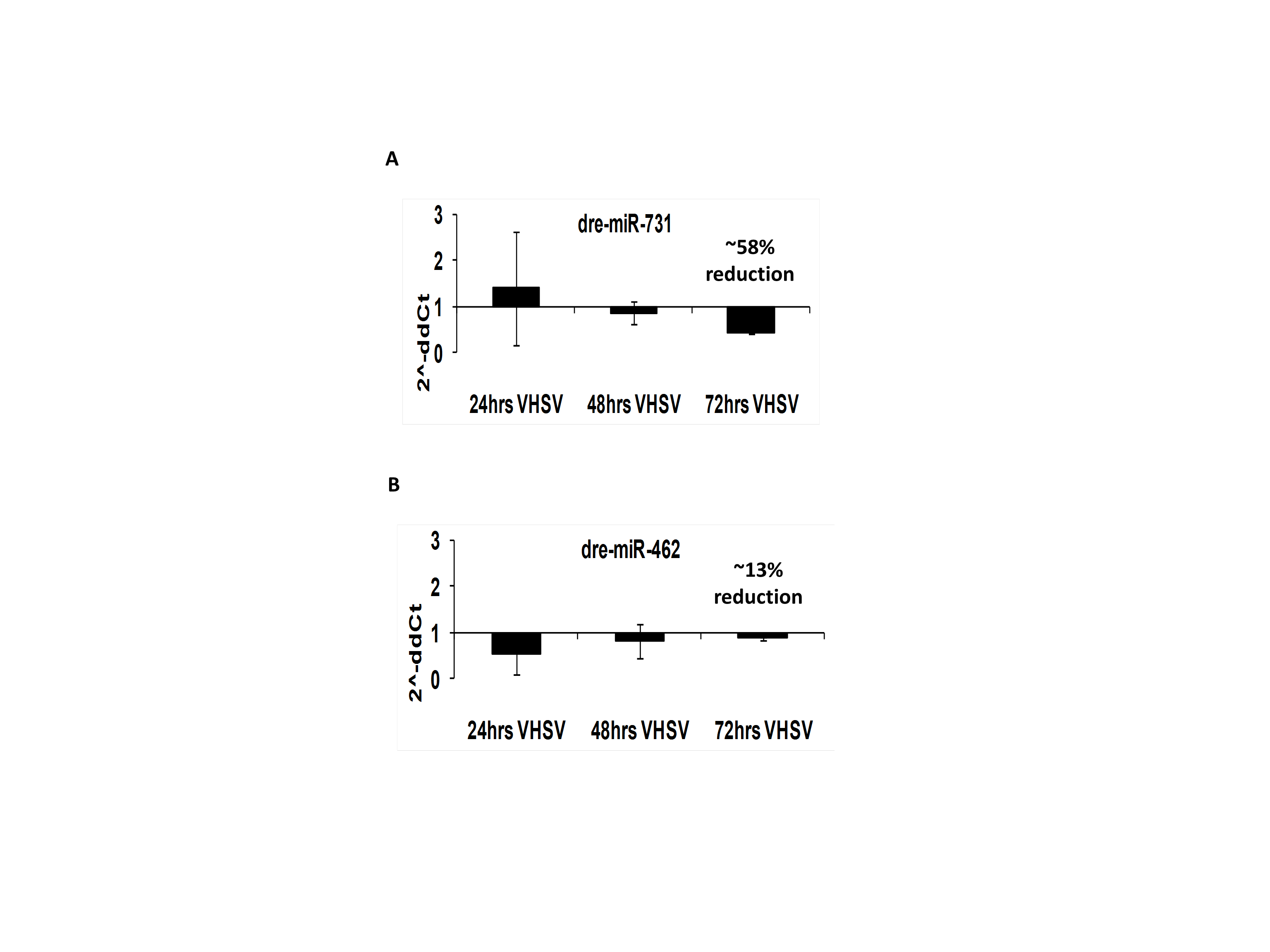

Supplement: S3 Fig — (TIF) [file pone.0132434.s003.tif]

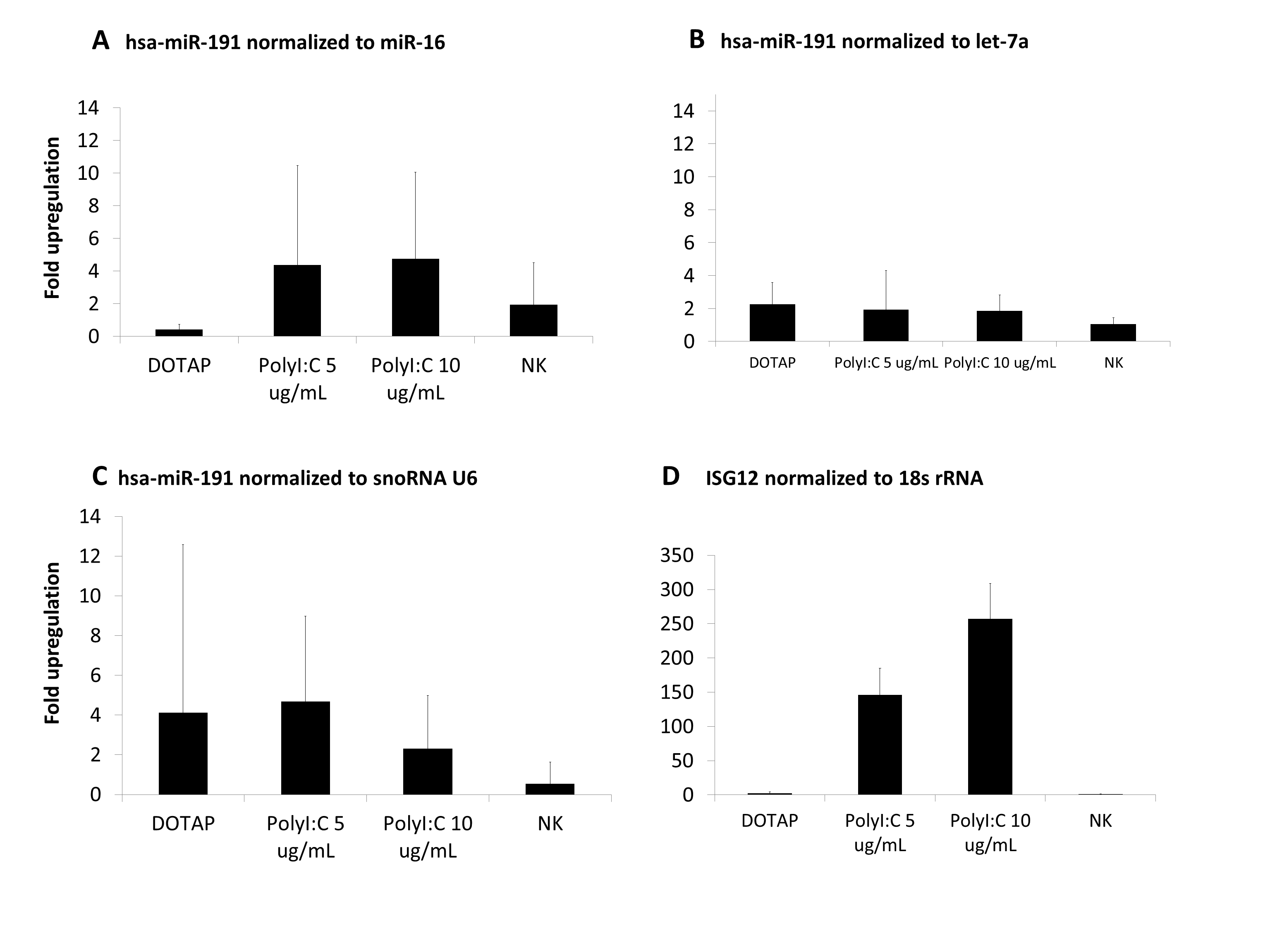

Supplement: S4 Fig — (A-C) Immune stimulation by poly I:C does not induce upregulation of miR-191 in HeLa cells. Normalization to hsa-miR-16 (A) showed a tendency towards regulation following poly I:C stimulation but was not significant. Normalization of the same expression data to hsa-let-7a (B) and hsa-snRNA U6 (C) did also not show any significant changes. Cells were either mock treated with DOTAP or treated with DOTAP-formulated poly I:C at 5 or 10 ug/ml concentrations. The negative control (NK) consisted of untreated cells. Standard deviations are shown. (D) Despite no significant upregulation of hsa-miR-191 following poly I:C stimulation, poly I:C treatment induced a strong concentration-dependent interferon response, as shown by the upregulation of ISG12 in Hela cells. Note that the values on the y-axis in (D) are much higher than in (A-C). The samples were also checked for the regulation of hsa-miR-425, which also showed no significant regulation (data not shown). Human embryo kidney cells (HEK293T) were used as negative control cells because an innate cellular response in these cells cannot be induced by poly I:C. Accordingly, these neither regulated ISG12 nor miR-191/miR-425 (data not shown). (TIF) [file pone.0132434.s004.tif]
