# Supplementary material for: Two Virus-Induced MicroRNAs Known Only from Teleost Fishes Are Orthologues of MicroRNAs Involved in Cell Cycle Control in Humans
Source: PLoS One. 2015 Jul 24;10(7):e0132434. doi: 10.1371/journal.pone.0132434 (PMC4514678; doi:10.1371/journal.pone.0132434)
Supplement: S1 Table — (DOCX) [file pone.0132434.s006.docx]

Table S1. Primers used in qPCR experiments.

| Primer name* | Accession number | Primer sequence (5’🡪 3’)^a^ |
| --- | --- | --- |
| dre-miR-462-5p | [MIMAT0001855](http://mirbase.org/cgi-bin/mature.pl?mature_acc=MIMAT0001855) | TAACGGAACCCATAATGCAGCT |
| dre-miR-731-5p | [MIMAT0003761](http://mirbase.org/cgi-bin/mature.pl?mature_acc=MIMAT0003761) | AATGACACGTTTTCTCCCGGATCG |
| omy-snoRNA U23 | AJ009730 | GCCCATGTCTGCTGTGAAACAAT |
| hsa-miR-191-5p | [MIMAT0000440](http://mirbase.org/cgi-bin/mature.pl?mature_acc=MIMAT0000440) | CAACGGAATCCCAAAAGCAGCTG |
| hsa-miR-425-5p | [MIMAT0003393](http://mirbase.org/cgi-bin/mature.pl?mature_acc=MIMAT0003393) | AATGACACGATCACTCCCGTTGA |
| hsa-miR-16-5p | [MIMAT0000069](http://mirbase.org/cgi-bin/mature.pl?mature_acc=MIMAT0000069) | TAGCAGCACGTAAATATTGGCG |
| hsa-let-7a | MIMAT0000062 | TGAGGTAGTAGGTTGTATAGTT |
| hsa-snRNA U6 | NR_004394.1 | CTCGCTTCGGCAGCACATATACT |
| hsa-ISG-12 | X67325.1 | CAAATTCTGCATCTCCAGAGG |
| hsa-18S rRNA | K03432.1 | TTCCGTAGGTGAACCTGCGGA |

*dre = Danio rerio (zebrafish); hsa = Homo sapiens (human); omy = *Oncorhynchus mykiss* (rainbow trout)

^a^ All primers are forward primers used together with a universal reverse primer supplied in the QuantiMir RT Kit as described in the Materials and Methods
