# Supplementary material for: Two Virus-Induced MicroRNAs Known Only from Teleost Fishes Are Orthologues of MicroRNAs Involved in Cell Cycle Control in Humans
Source: PLoS One. 2015 Jul 24;10(7):e0132434. doi: 10.1371/journal.pone.0132434 (PMC4514678; doi:10.1371/journal.pone.0132434)
Supplement: S2 Table — (Only top-ranked targets are shown.) (DOCX) [file pone.0132434.s007.docx]

**Table S2. Selected putative targets of miR-191 in vertebrate genomes predicted using the TargetScan Release 6.2 algorithm and ranked by their probability of conserved targeting (P_CT_). (Only top-ranked targets are shown.)**

| **Target Gene** | **Representative transcript** | **Gene name** | **Vertebrates whose miR-191have predicted targets in human mRNA orthologues** | | | | | | | | | | | | | | | |  |
| --- | --- | --- | --- | --- | --- | --- | --- | --- | --- | --- | --- | --- | --- | --- | --- | --- | --- | --- | --- |
|  |  |  |  |  |  | |  | |  | |  | |  | |  |  | |  | |
|  |  |  | **Rat** | **Frog** | | **Cow** | | **Opossum** | | **Rhesus** | | **Dog** | | **Chimpanzee** | **Mouse** | **Platypus** | **Horse** | |  |
| [**BDNF**](http://www.ncbi.nlm.nih.gov/sites/entrez?Db=gene&Cmd=ShowDetailView&TermToSearch=627) | [**NM_001143805**](http://www.ncbi.nlm.nih.gov/entrez/query.fcgi?cmd=Search&db=nuccore&term==NM_001143805) | brain-derived neurotrophic factor | + | + | | + | | + | | + | | + | | + | + | + | + | |  |
| [**CASK**](http://www.ncbi.nlm.nih.gov/sites/entrez?Db=gene&Cmd=ShowDetailView&TermToSearch=8573) | [**NM_001126054**](http://www.ncbi.nlm.nih.gov/entrez/query.fcgi?cmd=Search&db=nuccore&term==NM_001126054) | calcium/calmodulin-dependent serine protein kinase (MAGUK family) | + | + | | + | | + | | + | | + | | + | + | + | + | |  |
| [**TMOD2**](http://www.ncbi.nlm.nih.gov/sites/entrez?Db=gene&Cmd=ShowDetailView&TermToSearch=29767) | [**NM_001142885**](http://www.ncbi.nlm.nih.gov/entrez/query.fcgi?cmd=Search&db=nuccore&term==NM_001142885) | tropomodulin 2 (neuronal) | + | - | | - | | + | | + | | + | | + | + | - | + | |  |
| [**IFFO2**](http://www.ncbi.nlm.nih.gov/sites/entrez?Db=gene&Cmd=ShowDetailView&TermToSearch=126917) | [**NM_001136265**](http://www.ncbi.nlm.nih.gov/entrez/query.fcgi?cmd=Search&db=nuccore&term==NM_001136265) | intermediate filament family orphan 2 | + | - | | + | | - | | + | | + | | + | + | - | + | |  |
| [**NEURL4**](http://www.ncbi.nlm.nih.gov/sites/entrez?Db=gene&Cmd=ShowDetailView&TermToSearch=84461) | [**NM_001005408**](http://www.ncbi.nlm.nih.gov/entrez/query.fcgi?cmd=Search&db=nuccore&term==NM_001005408) | neuralized homolog 4 (Drosophila) | + | - | | + | | + | | + | | + | | + | + | - | - | |  |
| [**ZNF362**](http://www.ncbi.nlm.nih.gov/sites/entrez?Db=gene&Cmd=ShowDetailView&TermToSearch=149076) | [**NM_152493**](http://www.ncbi.nlm.nih.gov/entrez/query.fcgi?cmd=Search&db=nuccore&term==NM_152493) | zinc finger protein 362 | - | - | | + | | + | | + | | + | | + | - | + | + | |  |
| [**CDK6**](http://www.ncbi.nlm.nih.gov/sites/entrez?Db=gene&Cmd=ShowDetailView&TermToSearch=1021) | [**NM_001145306**](http://www.ncbi.nlm.nih.gov/entrez/query.fcgi?cmd=Search&db=nuccore&term==NM_001145306) | cyclin-dependent kinase 6 | - | - | | + | | - | | + | | + | | + | + | - | + | |  |
| [**MAPRE3**](http://www.ncbi.nlm.nih.gov/sites/entrez?Db=gene&Cmd=ShowDetailView&TermToSearch=22924) | [**NM_012326**](http://www.ncbi.nlm.nih.gov/entrez/query.fcgi?cmd=Search&db=nuccore&term==NM_012326) | microtubule-associated protein, RP/EB family, member 3 | + | - | | + | | + | | - | | + | | - | + | + | + | |  |
| [**RCC2**](http://www.ncbi.nlm.nih.gov/sites/entrez?Db=gene&Cmd=ShowDetailView&TermToSearch=55920) | [**NM_001136204**](http://www.ncbi.nlm.nih.gov/entrez/query.fcgi?cmd=Search&db=nuccore&term==NM_001136204) | regulator of chromosome condensation 2 | + | - | | - | | + | | + | | + | | + | - | - | + | |  |
| [**AMMECR1**](http://www.ncbi.nlm.nih.gov/sites/entrez?Db=gene&Cmd=ShowDetailView&TermToSearch=9949) | [**NM_001025580**](http://www.ncbi.nlm.nih.gov/entrez/query.fcgi?cmd=Search&db=nuccore&term==NM_001025580) | Alport syndrome, mental retardation, midface hypoplasia and elliptocytosis chromosomal region gene 1 | + | + | | + | | + | | + | | + | | + | + | + | + | |  |
| [**MSI1**](http://www.ncbi.nlm.nih.gov/sites/entrez?Db=gene&Cmd=ShowDetailView&TermToSearch=4440) | [**NM_002442**](http://www.ncbi.nlm.nih.gov/entrez/query.fcgi?cmd=Search&db=nuccore&term==NM_002442) | musashi homolog 1 (Drosophila) | - | - | | + | | - | | + | | + | | + | - | - | + | |  |
| [**NFIA**](http://www.ncbi.nlm.nih.gov/sites/entrez?Db=gene&Cmd=ShowDetailView&TermToSearch=4774) | [**NM_001134673**](http://www.ncbi.nlm.nih.gov/entrez/query.fcgi?cmd=Search&db=nuccore&term==NM_001134673) | nuclear factor I/A | + | + | | + | | + | | - | | + | | + | + | + | + | |  |
| [**WIZ**](http://www.ncbi.nlm.nih.gov/sites/entrez?Db=gene&Cmd=ShowDetailView&TermToSearch=58525) | [**NM_021241**](http://www.ncbi.nlm.nih.gov/entrez/query.fcgi?cmd=Search&db=nuccore&term==NM_021241) | widely interspaced zinc finger motifs | + | - | | - | | - | | + | | + | | + | + | - | + | |  |
| [**SLC12A5**](http://www.ncbi.nlm.nih.gov/sites/entrez?Db=gene&Cmd=ShowDetailView&TermToSearch=57468) | [**NM_001134771**](http://www.ncbi.nlm.nih.gov/entrez/query.fcgi?cmd=Search&db=nuccore&term==NM_001134771) | solute carrier family 12 (potassium/chloride transporter), member 5 | - | - | | - | | - | | + | | + | | + | + | - | + | |  |
| [**GAP43**](http://www.ncbi.nlm.nih.gov/sites/entrez?Db=gene&Cmd=ShowDetailView&TermToSearch=2596) | [**NM_001130064**](http://www.ncbi.nlm.nih.gov/entrez/query.fcgi?cmd=Search&db=nuccore&term==NM_001130064) | growth associated protein 43 | + | + | | + | | + | | + | | + | | + | + | + | + | |  |
| [**AJAP1**](http://www.ncbi.nlm.nih.gov/sites/entrez?Db=gene&Cmd=ShowDetailView&TermToSearch=55966) | [**NM_018836**](http://www.ncbi.nlm.nih.gov/entrez/query.fcgi?cmd=Search&db=nuccore&term==NM_018836) | adherens junctions associated protein 1 | - | - | | + | | + | | + | | + | | + | + | + | + | |  |
| [**FZD5**](http://www.ncbi.nlm.nih.gov/sites/entrez?Db=gene&Cmd=ShowDetailView&TermToSearch=7855) | [**NM_003468**](http://www.ncbi.nlm.nih.gov/entrez/query.fcgi?cmd=Search&db=nuccore&term==NM_003468) | frizzled family receptor 5 | + | - | | + | | - | | + | | + | | + | + | - | - | |  |
| [**ATP2B2**](http://www.ncbi.nlm.nih.gov/sites/entrez?Db=gene&Cmd=ShowDetailView&TermToSearch=491) | [**NM_001001331**](http://www.ncbi.nlm.nih.gov/entrez/query.fcgi?cmd=Search&db=nuccore&term==NM_001001331) | ATPase, Ca++ transporting, plasma membrane 2 | + | - | | + | | - | | + | | + | | + | + | - | + | |  |
| [**ZCCHC24**](http://www.ncbi.nlm.nih.gov/sites/entrez?Db=gene&Cmd=ShowDetailView&TermToSearch=219654) | [**NM_153367**](http://www.ncbi.nlm.nih.gov/entrez/query.fcgi?cmd=Search&db=nuccore&term==NM_153367) | zinc finger, CCHC domain containing 24 | + | - | | - | | - | | + | | + | | + | + | - | + | |  |
| [**RNF139**](http://www.ncbi.nlm.nih.gov/sites/entrez?Db=gene&Cmd=ShowDetailView&TermToSearch=11236) | [**NM_007218**](http://www.ncbi.nlm.nih.gov/entrez/query.fcgi?cmd=Search&db=nuccore&term==NM_007218) | ring finger protein 139 | + | - | | + | | + | | + | | + | | + | + | - | + | |  |
| [**FUBP3**](http://www.ncbi.nlm.nih.gov/sites/entrez?Db=gene&Cmd=ShowDetailView&TermToSearch=8939) | [**NM_003934**](http://www.ncbi.nlm.nih.gov/entrez/query.fcgi?cmd=Search&db=nuccore&term==NM_003934) | far upstream element (FUSE) binding protein 3 | - | + | | + | | + | | + | | + | | + | + | + | + | |  |
