# Supplementary material for: Two Virus-Induced MicroRNAs Known Only from Teleost Fishes Are Orthologues of MicroRNAs Involved in Cell Cycle Control in Humans
Source: PLoS One. 2015 Jul 24;10(7):e0132434. doi: 10.1371/journal.pone.0132434 (PMC4514678; doi:10.1371/journal.pone.0132434)
Supplement: S3 Table — (DOCX) [file pone.0132434.s008.docx]

**Table S3. Putative targets of miR-425 in vertebrate genomes predicted using the TargetScan Release 6.2 algorithm and ranked by their probability of conserved targeting (P_CT_).**

| **Target Gene** | **Representative transcript** | **Gene name** | **Vertebrates whose miR-425 have predicted targets in human mRNA orthologues** | | | | | | | | | |
| --- | --- | --- | --- | --- | --- | --- | --- | --- | --- | --- | --- | --- |
|  |  |  |  |  |  |  |  |  |  |  |  |  |
|  |  |  | **Rat** | **Frog** | **Cow** | **Opossum** | **Rhesus** | **Dog** | **Chimpanzee** | **Mouse** | **Platypus** | **Horse** |
| [**NUFIP2**](http://www.ncbi.nlm.nih.gov/sites/entrez?Db=gene&Cmd=ShowDetailView&TermToSearch=57532) | [**NM_020772**](http://www.ncbi.nlm.nih.gov/entrez/query.fcgi?cmd=Search&db=nuccore&term==NM_020772) | nuclear fragile X mental retardation protein interacting protein 2 | + | - | - | - | + | + | + | + | + | - |
| [**FOXJ3**](http://www.ncbi.nlm.nih.gov/sites/entrez?Db=gene&Cmd=ShowDetailView&TermToSearch=22887) | [**NM_001198850**](http://www.ncbi.nlm.nih.gov/entrez/query.fcgi?cmd=Search&db=nuccore&term==NM_001198850) | forkhead box J3 | + | + | - | - | + | + | + | + | + | - |
| [**C11orf41**](http://www.ncbi.nlm.nih.gov/sites/entrez?Db=gene&Cmd=ShowDetailView&TermToSearch=25758) | [**NM_012194**](http://www.ncbi.nlm.nih.gov/entrez/query.fcgi?cmd=Search&db=nuccore&term==NM_012194) | chromosome 11 open reading frame 41 | - | - | - | - | + | - | + | - | - | - |
| [**STRN**](http://www.ncbi.nlm.nih.gov/sites/entrez?Db=gene&Cmd=ShowDetailView&TermToSearch=6801) | [**NM_003162**](http://www.ncbi.nlm.nih.gov/entrez/query.fcgi?cmd=Search&db=nuccore&term==NM_003162) | striatin, calmodulin binding protein | - | - | - | - | + | + | + | - | - | - |
| [**ZAK**](http://www.ncbi.nlm.nih.gov/sites/entrez?Db=gene&Cmd=ShowDetailView&TermToSearch=51776) | [**NM_133646**](http://www.ncbi.nlm.nih.gov/entrez/query.fcgi?cmd=Search&db=nuccore&term==NM_133646) | sterile alpha motif and leucine zipper containing kinase AZK | - | - | - | - | - | + | + | - | + | - |
| [**ATP5G3**](http://www.ncbi.nlm.nih.gov/sites/entrez?Db=gene&Cmd=ShowDetailView&TermToSearch=518) | [**NM_001002258**](http://www.ncbi.nlm.nih.gov/entrez/query.fcgi?cmd=Search&db=nuccore&term==NM_001002258) | ATP synthase, H+ transporting, mitochondrial Fo complex, subunit C3 (subunit 9) | + | - | - | - | + | + | + | + | - | - |
| [**FAM135B**](http://www.ncbi.nlm.nih.gov/sites/entrez?Db=gene&Cmd=ShowDetailView&TermToSearch=51059) | [**NM_015912**](http://www.ncbi.nlm.nih.gov/entrez/query.fcgi?cmd=Search&db=nuccore&term==NM_015912) | family with sequence similarity 135, member B | + | - | - | - | + | + | + | + | - | - |
| [**CBL**](http://www.ncbi.nlm.nih.gov/sites/entrez?Db=gene&Cmd=ShowDetailView&TermToSearch=867) | [**NM_005188**](http://www.ncbi.nlm.nih.gov/entrez/query.fcgi?cmd=Search&db=nuccore&term==NM_005188) | Cas-Br-M (murine) ecotropic retroviral transforming sequence | - | - | - | - | + | + | + | - | + | - |
| [**WWC3**](http://www.ncbi.nlm.nih.gov/sites/entrez?Db=gene&Cmd=ShowDetailView&TermToSearch=55841) | [**NM_015691**](http://www.ncbi.nlm.nih.gov/entrez/query.fcgi?cmd=Search&db=nuccore&term==NM_015691) | WWC family member 3 | - | - | - | - | + | + | + | - | + | - |
| [**RALGAPA2**](http://www.ncbi.nlm.nih.gov/sites/entrez?Db=gene&Cmd=ShowDetailView&TermToSearch=57186) | [**NM_020343**](http://www.ncbi.nlm.nih.gov/entrez/query.fcgi?cmd=Search&db=nuccore&term==NM_020343) | Ral GTPase activating protein, alpha subunit 2 (catalytic) | - | - | - | - | + | - | + | - | - | - |
| [**DUSP16**](http://www.ncbi.nlm.nih.gov/sites/entrez?Db=gene&Cmd=ShowDetailView&TermToSearch=80824) | [**NM_030640**](http://www.ncbi.nlm.nih.gov/entrez/query.fcgi?cmd=Search&db=nuccore&term==NM_030640) | dual specificity phosphatase 16 | + | - | - | - | + | - | + | - | - | - |
| [**CSNK1G1**](http://www.ncbi.nlm.nih.gov/sites/entrez?Db=gene&Cmd=ShowDetailView&TermToSearch=53944) | [**NM_022048**](http://www.ncbi.nlm.nih.gov/entrez/query.fcgi?cmd=Search&db=nuccore&term==NM_022048) | casein kinase 1, gamma 1 | + | - | - | - | + | + | + | + | + | - |
| [**HLF**](http://www.ncbi.nlm.nih.gov/sites/entrez?Db=gene&Cmd=ShowDetailView&TermToSearch=3131) | [**NM_002126**](http://www.ncbi.nlm.nih.gov/entrez/query.fcgi?cmd=Search&db=nuccore&term==NM_002126) | hepatic leukemia factor | - | - | - | - | - | + | + | - | - | - |
| [**FBXO41**](http://www.ncbi.nlm.nih.gov/sites/entrez?Db=gene&Cmd=ShowDetailView&TermToSearch=150726) | [**NM_001080410**](http://www.ncbi.nlm.nih.gov/entrez/query.fcgi?cmd=Search&db=nuccore&term==NM_001080410) | F-box protein 41 | - | - | - | - | + | + | + | - | - | - |
| [**KLF3**](http://www.ncbi.nlm.nih.gov/sites/entrez?Db=gene&Cmd=ShowDetailView&TermToSearch=51274) | [**NM_016531**](http://www.ncbi.nlm.nih.gov/entrez/query.fcgi?cmd=Search&db=nuccore&term==NM_016531) | Kruppel-like factor 3 (basic) | + | - | - | - | + | + | + | - | - | - |
| [**ANTXR1**](http://www.ncbi.nlm.nih.gov/sites/entrez?Db=gene&Cmd=ShowDetailView&TermToSearch=84168) | [**NM_032208**](http://www.ncbi.nlm.nih.gov/entrez/query.fcgi?cmd=Search&db=nuccore&term==NM_032208) | anthrax toxin receptor 1 | - | - | - | - | + | - | + | + | - | - |
| [**FAM133B**](http://www.ncbi.nlm.nih.gov/sites/entrez?Db=gene&Cmd=ShowDetailView&TermToSearch=257415) | [**NM_001040057**](http://www.ncbi.nlm.nih.gov/entrez/query.fcgi?cmd=Search&db=nuccore&term==NM_001040057) | family with sequence similarity 133, member B | - | - | - | - | + | + | + | - | - | - |
| [**IGF1**](http://www.ncbi.nlm.nih.gov/sites/entrez?Db=gene&Cmd=ShowDetailView&TermToSearch=3479) | [**NM_000618**](http://www.ncbi.nlm.nih.gov/entrez/query.fcgi?cmd=Search&db=nuccore&term==NM_000618) | insulin-like growth factor 1 (somatomedin C) | + | - | - | - | + | - | + | + | - | - |
| [**TLK1**](http://www.ncbi.nlm.nih.gov/sites/entrez?Db=gene&Cmd=ShowDetailView&TermToSearch=9874) | [**NM_001136554**](http://www.ncbi.nlm.nih.gov/entrez/query.fcgi?cmd=Search&db=nuccore&term==NM_001136554) | tousled-like kinase 1 | - | - | - | - | + | - | + | - | + | - |
| [**MPP5**](http://www.ncbi.nlm.nih.gov/sites/entrez?Db=gene&Cmd=ShowDetailView&TermToSearch=64398) | [**NM_022474**](http://www.ncbi.nlm.nih.gov/entrez/query.fcgi?cmd=Search&db=nuccore&term==NM_022474) | membrane protein, palmitoylated 5 (MAGUK p55 subfamily member 5) | - | - | - | - | + | + | + | - | - | - |
| [**CPLX2**](http://www.ncbi.nlm.nih.gov/sites/entrez?Db=gene&Cmd=ShowDetailView&TermToSearch=10814) | [**NM_001008220**](http://www.ncbi.nlm.nih.gov/entrez/query.fcgi?cmd=Search&db=nuccore&term==NM_001008220) | complexin 2 | - | - | - | - | - | + | + | - | - | - |
