# Supplementary material for: Two Virus-Induced MicroRNAs Known Only from Teleost Fishes Are Orthologues of MicroRNAs Involved in Cell Cycle Control in Humans
Source: PLoS One. 2015 Jul 24;10(7):e0132434. doi: 10.1371/journal.pone.0132434 (PMC4514678; doi:10.1371/journal.pone.0132434)
Supplement: S4 Table — (DOCX) [file pone.0132434.s009.docx]

**Table S4. Putative targets of miR-462 in the zebrafish genome predicted using the TargetScanFish Release 6.2 algorithm and ranked by their probability of conserved targeting (P_CT_).**

| **Target gene** | **Representative 3’ UTR** | **Transcript name** |
| --- | --- | --- |
| [**si:ch211-241b2.1**](http://useast.ensembl.org/Danio_rerio/Gene/Variation_Gene/Table?g=ENSDARG00000044845) | ENSDARG00000044845.1 | Novel protein annotated by Havana |
| **[IL1RAP](http://useast.ensembl.org/Danio_rerio/Gene/Variation_Gene/Table?g=ENSDARG00000091088)** | ENSDARG00000091088.1 | uncharacterized protein |
| [**TRAF2 (1 of 2)**](http://useast.ensembl.org/Danio_rerio/Gene/Variation_Gene/Table?g=ENSDARG00000078378) | ENSDARG00000078378.1 | TNF receptor-associated factor 2 |
| [**CABZ01038499.1**](http://useast.ensembl.org/Danio_rerio/Gene/Variation_Gene/Table?g=ENSDARG00000088258) | ENSDARG00000088258.1 | chromosome 16 open reading frame 52 |
| [**ABTB1**](http://useast.ensembl.org/Danio_rerio/Gene/Variation_Gene/Table?g=ENSDARG00000063354) | ENSDARG00000063354.1 | ankyrin repeat and BTB (POZ) domain containing 1 |
| **[anks1b](http://useast.ensembl.org/Danio_rerio/Gene/Variation_Gene/Table?g=ENSDARG00000003512)** | ENSDARG00000003512.1 | ankyrin repeat and sterile alpha motif domain containing 1B |
| **[ampd2](http://useast.ensembl.org/Danio_rerio/Gene/Variation_Gene/Table?g=ENSDARG00000029952)** | ENSDARG00000029952.1 | adenosine monophosphate deaminase 2 (isoform L) |
| **[CABZ01045212.1](http://useast.ensembl.org/Danio_rerio/Gene/Variation_Gene/Table?g=ENSDARG00000087525)** | ENSDARG00000087525.1 | [Uncharacterized protein](http://www.uniprot.org/uniprot/?query=E7FA68&sort=score) |
| [**fermt2**](http://useast.ensembl.org/Danio_rerio/Gene/Variation_Gene/Table?g=ENSDARG00000020242) | ENSDARG00000020242.1 | fermitin family homolog 2 (Drosophila) |
| [**cdk12**](http://useast.ensembl.org/Danio_rerio/Gene/Variation_Gene/Table?g=ENSDARG00000063726) | ENSDARG00000063726.1 | cyclin-dependent kinase 12 |
| [**elp3**](http://useast.ensembl.org/Danio_rerio/Gene/Variation_Gene/Table?g=ENSDARG00000042005) | ENSDARG00000042005.1 | elongation protein 3 homolog (S. cerevisiae) |
| **[arntl1b](http://useast.ensembl.org/Danio_rerio/Gene/Variation_Gene/Table?g=ENSDARG00000035732)** | ENSDARG00000035732.1 | aryl hydrocarbon receptor nuclear translocator-like 1b |
| **[adcy7](http://useast.ensembl.org/Danio_rerio/Gene/Variation_Gene/Table?g=ENSDARG00000060070)** | ENSDARG00000060070.1 | adenylate cyclase 7 |
| [**SNX19 (1 of 2)**](http://useast.ensembl.org/Danio_rerio/Gene/Variation_Gene/Table?g=ENSDARG00000079931) | ENSDARG00000079931.1 | Uncharacterized protein |
| [**SGSM2**](http://useast.ensembl.org/Danio_rerio/Gene/Variation_Gene/Table?g=ENSDARG00000063307) | ENSDARG00000063307.1 | small G protein signaling modulator 2 |
| **[ZNF507](http://useast.ensembl.org/Danio_rerio/Gene/Variation_Gene/Table?g=ENSDARG00000052164)** | ENSDARG00000052164.1 | Uncharacterized protein |
| [**pex19**](http://useast.ensembl.org/Danio_rerio/Gene/Variation_Gene/Table?g=ENSDARG00000004891) | ENSDARG00000004891.1 | peroxisomal biogenesis factor 19 |
| [**tgfb1a**](http://useast.ensembl.org/Danio_rerio/Gene/Variation_Gene/Table?g=ENSDARG00000041502) | ENSDARG00000041502.1 | transforming growth factor, beta 1a |
| **[fam69b](http://useast.ensembl.org/Danio_rerio/Gene/Variation_Gene/Table?g=ENSDARG00000059881)** | ENSDARG00000059881.1 | family with sequence similarity 69, member B |
| **[ZEB1 (2 of 2)](http://useast.ensembl.org/Danio_rerio/Gene/Variation_Gene/Table?g=ENSDARG00000016788)** | ENSDARG00000016788.1 | zinc finger E-box binding homeobox 1a |
| **[ERBB4 (3 of 5)](http://useast.ensembl.org/Danio_rerio/Gene/Variation_Gene/Table?g=ENSDARG00000090408)** | ENSDARG00000090408.1 | v-erb-b2 avian erythroblastic leukemia viral oncogene homolog 4 |
| **[uhmk1](http://useast.ensembl.org/Danio_rerio/Gene/Variation_Gene/Table?g=ENSDARG00000059575)** | ENSDARG00000059575.1 | U2AF homology motif (UHM) kinase 1 |
| **[scpp1](http://useast.ensembl.org/Danio_rerio/Gene/Variation_Gene/Table?g=ENSDARG00000090416)** | ENSDARG00000090416.1 | secretory calcium-binding phosphoprotein 1 |
| [**CU856539.3**](http://useast.ensembl.org/Danio_rerio/Gene/Variation_Gene/Table?g=ENSDARG00000058949) | ENSDARG00000058949.1 | spindlin family, member 2B |
| [**sall1a**](http://useast.ensembl.org/Danio_rerio/Gene/Variation_Gene/Table?g=ENSDARG00000074319) | ENSDARG00000074319.1 | sal-like 1a (Drosophila) |
| [**nbeal2**](http://useast.ensembl.org/Danio_rerio/Gene/Variation_Gene/Table?g=ENSDARG00000057508) | ENSDARG00000057508.1 | neurobeachin-like 2 |
| [**mtx3**](http://useast.ensembl.org/Danio_rerio/Gene/Variation_Gene/Table?g=ENSDARG00000030390) | ENSDARG00000030390.1 | metaxin 3 |
| [**mtx3**](http://useast.ensembl.org/Danio_rerio/Gene/Variation_Gene/Table?g=ENSDARG00000090195) | ENSDARG00000090195.1 | metaxin 3 |
| [**C1H4orf27**](http://useast.ensembl.org/Danio_rerio/Gene/Variation_Gene/Table?g=ENSDARG00000057114) | ENSDARG00000057114.1 | chromosome 4 open reading frame 27 |
| [**mxd**](http://useast.ensembl.org/Danio_rerio/Gene/Variation_Gene/Table?g=ENSDARG00000023369) | ENSDARG00000023369.1 | myxovirus (influenza virus) resistance D |
| **[gba2](http://useast.ensembl.org/Danio_rerio/Gene/Variation_Gene/Table?g=ENSDARG00000061472)** | ENSDARG00000061472.1 | glucosidase, beta (bile acid) 2 |
| [**psmb5**](http://useast.ensembl.org/Danio_rerio/Gene/Variation_Gene/Table?g=ENSDARG00000075445) | ENSDARG00000075445.1 | proteasome (prosome, macropain) subunit, beta type, 5 |
| **[bckdha](http://useast.ensembl.org/Danio_rerio/Gene/Variation_Gene/Table?g=ENSDARG00000040555)** | ENSDARG00000040555.1 | branched chain keto acid dehydrogenase E1, alpha polypeptide |
| **[kbtbd5](http://useast.ensembl.org/Danio_rerio/Gene/Variation_Gene/Table?g=ENSDARG00000039052)** | ENSDARG00000039052.1 | kelch-like 40a (Drosophila) |
| [**C5H9orf86 (2 of 2)**](http://useast.ensembl.org/Danio_rerio/Gene/Variation_Gene/Table?g=ENSDARG00000079046) | ENSDARG00000079046.1 | RAB, member RAS oncogene family-like 6 |
| **[FP236334.1](http://useast.ensembl.org/Danio_rerio/Gene/Variation_Gene/Table?g=ENSDARG00000095664)** | ENSDARG00000095664.1 | [Uncharacterized protein](http://www.uniprot.org/uniprot/?query=F1QLZ6&sort=score) |
| [**lamp2**](http://useast.ensembl.org/Danio_rerio/Gene/Variation_Gene/Table?g=ENSDARG00000014914) | ENSDARG00000014914.1 | lysosomal membrane glycoprotein 2 |
| [**CR407701.1**](http://useast.ensembl.org/Danio_rerio/Gene/Variation_Gene/Table?g=ENSDARG00000089094) | ENSDARG00000089094.1 | [Uncharacterized protein](http://www.uniprot.org/uniprot/?query=E7FEV2&sort=score) |
| [**tpd52**](http://useast.ensembl.org/Danio_rerio/Gene/Variation_Gene/Table?g=ENSDARG00000061713) | ENSDARG00000061713.1 | tumor protein D52 |
| [**tmem2**](http://useast.ensembl.org/Danio_rerio/Gene/Variation_Gene/Table?g=ENSDARG00000061600) | ENSDARG00000061600.1 | transmembrane protein 2 |
| [**zbtb22b**](http://useast.ensembl.org/Danio_rerio/Gene/Variation_Gene/Table?g=ENSDARG00000003251) | ENSDARG00000003251.1 | zinc finger and BTB domain containing 22b |
| **[arl3l1](http://useast.ensembl.org/Danio_rerio/Gene/Variation_Gene/Table?g=ENSDARG00000028846)** | ENSDARG00000028846.1 | ADP-ribosylation factor-like 3, like 1 |
