# Supplementary material for: Two Virus-Induced MicroRNAs Known Only from Teleost Fishes Are Orthologues of MicroRNAs Involved in Cell Cycle Control in Humans
Source: PLoS One. 2015 Jul 24;10(7):e0132434. doi: 10.1371/journal.pone.0132434 (PMC4514678; doi:10.1371/journal.pone.0132434)
Supplement: S5 Table — (DOCX) [file pone.0132434.s010.docx]

**Table S5. Putative targets of miR-731 in the zebrafish genome predicted using the TargetScanFish Release 6.2 algorithm and ranked by their probability of conserved targeting (P_CT_).**

| **Target Gene** | **Representative 3’ UTR** | **Transcript name** |
| --- | --- | --- |
|  |  |  |
| [**NBL1**](http://useast.ensembl.org/Danio_rerio/Gene/Variation_Gene/Table?g=ENSDARG00000031898) | ENSDARG00000031898.1 | neuroblastoma 1, DAN family BMP antagonist |
| [**pfdn5**](http://useast.ensembl.org/Danio_rerio/Gene/Variation_Gene/Table?g=ENSDARG00000035043) | ENSDARG00000035043.1 | prefoldin 5 |
| [**ANK1 (1 of 2)\|ank1**](http://useast.ensembl.org/Danio_rerio/Gene/Variation_Gene/Table?g=ENSDARG059093\|092143) | ENSDARG059093\|092143.1 | not present in the current release of the Ensembl database |
| [**rab23**](http://useast.ensembl.org/Danio_rerio/Gene/Variation_Gene/Table?g=ENSDARG00000004151) | ENSDARG00000004151.1 | RAB23, member RAS oncogene family |
| [**UBAC2 (1 of 2)**](http://useast.ensembl.org/Danio_rerio/Gene/Variation_Gene/Table?g=ENSDARG00000060581) | ENSDARG00000060581.1 | UBA domain containing 2 |
| [**CABZ01045212.1**](http://useast.ensembl.org/Danio_rerio/Gene/Variation_Gene/Table?g=ENSDARG00000087525) | ENSDARG00000087525.1 | Uncharacterized protein |
| [**pob**](http://useast.ensembl.org/Danio_rerio/Gene/Variation_Gene/Table?g=ENSDARG00000020607) | ENSDARG00000020607.1 | ER membrane protein complex subunit 3 |
| [**XPR1 (1 of 2)**](http://useast.ensembl.org/Danio_rerio/Gene/Variation_Gene/Table?g=ENSDARG00000062449) | ENSDARG00000062449.1 | xenotropic and polytropic retrovirus receptor 1 |
| [**si:dkeyp-120h9.1**](http://useast.ensembl.org/Danio_rerio/Gene/Variation_Gene/Table?g=ENSDARG00000020278) | ENSDARG00000020278.1 | si:dkeyp-120h9.1 |
| [**BX248318.1**](http://useast.ensembl.org/Danio_rerio/Gene/Variation_Gene/Table?g=ENSDARG00000022570) | ENSDARG00000022570.1 | Uncharacterized protein |
| [**shpk**](http://useast.ensembl.org/Danio_rerio/Gene/Variation_Gene/Table?g=ENSDARG00000002355) | ENSDARG00000002355.1 | sedoheptulokinase |
| [**cdc42l2**](http://useast.ensembl.org/Danio_rerio/Gene/Variation_Gene/Table?g=ENSDARG00000057869) | ENSDARG00000057869.1 | cell division cycle 42 like 2 |
| [**dcp1a**](http://useast.ensembl.org/Danio_rerio/Gene/Variation_Gene/Table?g=ENSDARG00000003323) | ENSDARG00000003323.1 | DCP1 decapping enzyme homolog A (S. cerevisiae) |
| [**gpm6aa**](http://useast.ensembl.org/Danio_rerio/Gene/Variation_Gene/Table?g=ENSDARG00000055455) | ENSDARG00000055455.1 | glycoprotein M6Aa |
| [**UBAC2 (2 of 2)**](http://useast.ensembl.org/Danio_rerio/Gene/Variation_Gene/Table?g=ENSDARG00000093065) | ENSDARG00000093065.1 | UBA domain containing 2 |
| [**CABZ01079302.1**](http://useast.ensembl.org/Danio_rerio/Gene/Variation_Gene/Table?g=ENSDARG00000091604) | ENSDARG00000091604.1 | Uncharacterized protein |
| [**VAMP5**](http://useast.ensembl.org/Danio_rerio/Gene/Variation_Gene/Table?g=ENSDARG00000068262) | ENSDARG00000068262.1 | vesicle-associated membrane protein 5 |
| [**ldb2a**](http://useast.ensembl.org/Danio_rerio/Gene/Variation_Gene/Table?g=ENSDARG00000019579) | ENSDARG00000019579.1 | LIM-domain binding factor 2a |
| [**whsc1l1**](http://useast.ensembl.org/Danio_rerio/Gene/Variation_Gene/Table?g=ENSDARG00000062765) | ENSDARG00000062765.1 | Wolf-Hirschhorn syndrome candidate 1-like 1 |
| [**apeh**](http://useast.ensembl.org/Danio_rerio/Gene/Variation_Gene/Table?g=ENSDARG00000008703) | ENSDARG00000008703.1 | acylpeptide hydrolase |
| [**si:ch211-22n13.1**](http://useast.ensembl.org/Danio_rerio/Gene/Variation_Gene/Table?g=ENSDARG00000070528) | ENSDARG00000070528.1 | calcium channel, voltage-dependent, T type, alpha 1H subunit b |
| [**lipea**](http://useast.ensembl.org/Danio_rerio/Gene/Variation_Gene/Table?g=ENSDARG00000063037) | ENSDARG00000063037.1 | lipase, hormone-sensitive a |
| [**si:ch211-124k10.1**](http://useast.ensembl.org/Danio_rerio/Gene/Variation_Gene/Table?g=ENSDARG00000068731) | ENSDARG00000068731.1 | relaxin/insulin-like family peptide receptor 2, like |
| [**IPO11**](http://useast.ensembl.org/Danio_rerio/Gene/Variation_Gene/Table?g=ENSDARG00000054224) | ENSDARG00000054224.1 | importin 11 |
| [**cttnbp2nl**](http://useast.ensembl.org/Danio_rerio/Gene/Variation_Gene/Table?g=ENSDARG00000056091) | ENSDARG00000056091.1 | CTTNBP2 N-terminal like |
| [**onecutl**](http://useast.ensembl.org/Danio_rerio/Gene/Variation_Gene/Table?g=ENSDARG00000040253) | ENSDARG00000040253.1 | one cut domain, family member, like |
| [**smtnl1**](http://useast.ensembl.org/Danio_rerio/Gene/Variation_Gene/Table?g=ENSDARG00000041257) | ENSDARG00000041257.1 | smoothelin-like 1 |
| [**bcas3**](http://useast.ensembl.org/Danio_rerio/Gene/Variation_Gene/Table?g=ENSDARG00000090764) | ENSDARG00000090764.1 | breast carcinoma amplified sequence 3 |
| [**FUT11**](http://useast.ensembl.org/Danio_rerio/Gene/Variation_Gene/Table?g=ENSDARG00000057727) | ENSDARG00000057727.1 | fucosyltransferase 11 (alpha (1,3) fucosyltransferase) |
| [**rab34b**](http://useast.ensembl.org/Danio_rerio/Gene/Variation_Gene/Table?g=ENSDARG00000010977) | ENSDARG00000010977.1 | RAB34, member RAS oncogene family b |
| [**mpg**](http://useast.ensembl.org/Danio_rerio/Gene/Variation_Gene/Table?g=ENSDARG00000069729) | ENSDARG00000069729.1 | N-methylpurine-DNA glycosylase |
| [**slc4a4a**](http://useast.ensembl.org/Danio_rerio/Gene/Variation_Gene/Table?g=ENSDARG00000013730) | ENSDARG00000013730.1 | solute carrier family 4, member 4a |
| [**ccdc6a**](http://useast.ensembl.org/Danio_rerio/Gene/Variation_Gene/Table?g=ENSDARG00000043334) | ENSDARG00000043334.1 | coiled-coil domain containing 6a |
| [**fam155a**](http://useast.ensembl.org/Danio_rerio/Gene/Variation_Gene/Table?g=ENSDARG00000075858) | ENSDARG00000075858.1 | family with sequence similarity 155, member A |
| [**erbb4a**](http://useast.ensembl.org/Danio_rerio/Gene/Variation_Gene/Table?g=ENSDARG00000063207) | ENSDARG00000063207.1 | v-erb-a erythroblastic leukemia viral oncogene homolog 4a (avian) |
| [**si:ch211-217k17.10**](http://useast.ensembl.org/Danio_rerio/Gene/Variation_Gene/Table?g=ENSDARG00000079345) | ENSDARG00000079345.1 | si:ch211-217k17.10 |
| [**tom1**](http://useast.ensembl.org/Danio_rerio/Gene/Variation_Gene/Table?g=ENSDARG00000043638) | ENSDARG00000043638.1 | target of myb1 (chicken) |
| [**CR848759.2**](http://useast.ensembl.org/Danio_rerio/Gene/Variation_Gene/Table?g=ENSDARG00000079119) | ENSDARG00000079119.1 | si:ch211-229d2.5 |
| [**PACS1 (1 of 3)**](http://useast.ensembl.org/Danio_rerio/Gene/Variation_Gene/Table?g=ENSDARG00000019800) | ENSDARG00000019800.1 | phosphofurin acidic cluster sorting protein 1 |
| [**birc7**](http://useast.ensembl.org/Danio_rerio/Gene/Variation_Gene/Table?g=ENSDARG00000058082) | ENSDARG00000058082.1 | baculoviral IAP repeat-containing 7 |
| [**dnaja2l**](http://useast.ensembl.org/Danio_rerio/Gene/Variation_Gene/Table?g=ENSDARG00000010745) | ENSDARG00000010745.1 | DnaJ (Hsp40) homolog, subfamily A, member 2, like |
